# Sleep deprivation affects memory function, depression and anxiety-like behaviours in rats and mice: a systematic review and meta-analysis

**DOI:** 10.1093/braincomms/fcaf309

**Published:** 2025-08-28

**Authors:** Xiaofan Zhang, Cheng-wei Liu, Xin Sheng, Yifan Jiang, Sheng Zhang, XiaoYan Mo, Yuan Yang, Fengfei Ding

**Affiliations:** Department of Pharmacology, School of Basic Medical Sciences, Shanghai Medical College, Fudan University, Shanghai 200032, China; Department of Neurology, Tongji Hospital, Tongji Medical College, Huazhong University of Science and Technology, Wuhan 430030, China; Hubei Key Laboratory of Neural Injury and Functional Reconstruction, Huazhong University of Science and Technology Wuhan 430030, China; Department of Pharmacology, School of Basic Medical Sciences, Shanghai Medical College, Fudan University, Shanghai 200032, China; Department of Neurology, Tongji Hospital, Tongji Medical College, Huazhong University of Science and Technology, Wuhan 430030, China; Department of Neurology, Tongji Hospital, Tongji Medical College, Huazhong University of Science and Technology, Wuhan 430030, China; Center for Rehabilitation Medicine, Department of Neurology, Provincial People's Hospital, People's Hospital of Hangzhou Medical College, Hangzhou 310014, China; Department of Rehabilitation Medicine, The Second People's Hospital of Kunming, Rehabilitation Hospital Affiliated to Kunming University, Kunming 650021, China; Department of Neurology, Tongji Hospital, Tongji Medical College, Huazhong University of Science and Technology, Wuhan 430030, China; Hubei Key Laboratory of Neural Injury and Functional Reconstruction, Huazhong University of Science and Technology Wuhan 430030, China; Department of Pharmacology, School of Basic Medical Sciences, Shanghai Medical College, Fudan University, Shanghai 200032, China

**Keywords:** sleep deprivation, memory, depression, anxiety, species

## Abstract

Sleep deprivation paradigms have been employed in rat and mouse models to elucidate the function of sleep. The effects of sleep deprivation on memory function, as well as changes in depression- and anxiety-like behaviours, have been extensively investigated; however, the findings have often been inconsistent. In the present study, we conducted a comprehensive literature review of researches utilizing sleep deprivation paradigms in both rats and mice. A total of 164 original studies were analysed to extract results from behavioural tests concerning memory function and depression- and anxiety-like behaviours in wild-type rats or mice before and after sleep deprivation. The meta-analysis revealed that sleep deprivation consistently impaired memory function, irrespective of the paradigms, durations and species involved [*P* = 0.000, SMD (standardized mean difference) 95% CI (confidence intervals at 95%): −0.73 (−0.89, −0.57) for sleep deprivation; *P* = 0.000, SMD (95% CI): −0.75 (−0.93, −0.57) for rapid eye movement sleep deprivation]. Similar, albeit less pronounced, effects were observed on depression-like behaviours [*P* = 0.000, SMD (95% CI): −0.41 (−0.52, −0.29) for sleep deprivation; *P* = 0.000, SMD (95% CI): −0.60 (−0.79, −0.42) for rapid eye movement sleep deprivation]. The impact of sleep deprivation on anxiety-like behaviours was more variable. When considering both mice and rats, sleep deprivation generally exhibited anxiogenic effects [*P* = 0.049, SMD (95% CI): −0.19 (−0.39, −0.00) for sleep deprivation; *P* = 0.705, SMD (95% CI): 0.04 (−0.18, 0.27) for rapid eye movement sleep deprivation]. However, subgroup analyses indicated that rodent species and sleep durations demonstrated distinct responses to sleep deprivation. This study provides critical insights for selecting optimal paradigms, durations, species and behavioural tests in experimental designs.

## Teaser

Zhang *et al*. report that sleep deprivation consistently impairs memory function but generates diverse effects on depression and anxiety-like behaviours due to factors such as species, sex, age, or interventional paradigms, etc.

## Graphical Abstract

**Graphical Abstract:**
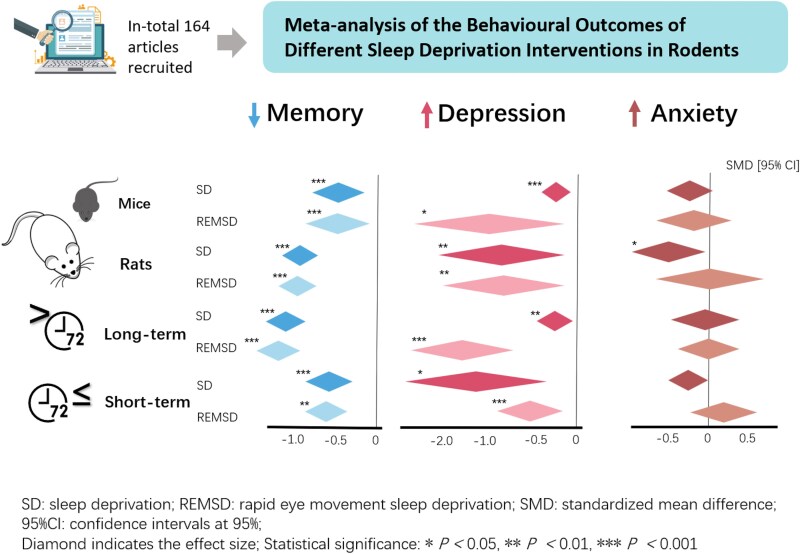

## Introduction

Sleep is a fundamental biological process that is highly conserved across various species. The mechanisms and functions of sleep remain subjects of active investigation, with numerous scientific questions still unanswered.^1^ In humans, rapid eye movement (REM) sleep and non-rapid eye movement (NREM) sleep occur in five to six periodic episodes throughout the course of a single night, each serving distinct physiological functions.^2^ Although the circadian rhythms in response to light are reversed in rodents such as rats or mice compared with humans, both NREM and REM sleep exhibit remarkable conservation between these groups.^3^ Experimental protocols for sleep deprivation (SD), which simulate sleep loss or restriction, have been extensively utilized to unravel the complexities of sleep.^4,5^ Commonly employed paradigms for sleep manipulation include total sleep deprivation (TSD), which keeps animals awake for a specified duration; rapid eye movement sleep deprivation (REMSD), which primarily suppresses REM sleep; and sleep fragmentation (SF), which induces intermittent awakenings during sleep periods.^6-8^

In healthy individuals experiencing SD, impaired memory, as well as symptoms of anxiety and depression, are commonly observed, which attract the attention of clinicians.^9-11^ This area of research is rapidly expanding, focusing on the functions of sleep and the effects of sleep insufficiency on behavioural outcomes.^12^ However, clinical studies often rely on self-reported questionnaires completed by insomnia patients to assess sleep loss, and they typically implemented acute, short-term interventions for SD or restriction in healthy individuals for ethical considerations.^13-15^ Animal models play crucial roles in elucidating the pathophysiological mechanisms underlying SD. Specifically, these models can be utilized for acute, chronic or selective SD, particularly in the context of REMSD.^16-18^

In recent decades, the volume of research on SD has increased dramatically revealing a wide array of behavioural outcomes in response to similar SD paradigms.^19-21^ These discrepancies may be attributed to variations in experimental paradigms, modelling durations and the species of animals utilized in SD studies. Additionally, the differences in SD protocols and behavioural assessments conducted by various researchers under diverse conditions may lead to divergent conclusions, potentially arising from systematic errors. Factors such as room temperature, humidity, seasonal variations and the sex of the rats and mice, among numerous others, may significantly influence the results of behavioural tests.^22-24^

In this study, we conducted a comprehensive literature review of experimental studies employing SD paradigms in rat or mouse models. TSD and SF can reduce or disrupt both NREM and REM sleep. Given that REM sleep can only occur following deep NREM sleep, disturbances in NREM sleep inevitably impact REM sleep. REMSD is a straightforward method that predominantly suppresses REM sleep. In this review, we categorized the studies utilizing REMSD alongside other methods to summarize the behavioural outcomes associated with REMSD and mixed studies that disrupt both REM and NREM sleep. Initially, we performed a global analysis to summarize the behavioural outcomes of experimental SD on memory function, as well as depression- and anxiety-like behaviours in wild-type adult rats and mice under both REMSD and SD (including all paradigms). Furthermore, we conducted subgroup analyses of REMSD and SD protocols to evaluate the isolated effects of species (rats versus mice) and experimental duration (short-term versus long-term protocols) on various behavioural paradigms. We also discussed potential confounding factors that may bias the behavioural outcomes. Finally, we compared the effect sizes of typical parameters from the behavioural tests to identify those with optimal detecting performance.

## Materials and methods

### Ethics approval and consent to participate

Not applicable.

### Search strategy

A comprehensive search of studies using experiments on SD was conducted using three databases: Pubmed, Scopus and Embase. The search strategy employed in PubMed was as follows: (((‘Sleep Deprivation”[Mesh]) OR (sleep restriction)) AND (animal [Filter])) AND (rodent OR rat OR mice OR mouse) AND (animal [Filter])). Filters applied included Other Animals and English language. Experimental SD was defined as housing the animals under specific conditions or in SD apparatuses, while control animals were maintained in a standard feeding environment. The search strategies utilized in Scopus and Embase are detailed in Supplementary Table 1. All types of literature published prior to July 2023 were included. Articles were selected through a two-round process. In the first round, titles and abstracts were screened for relevance to the study's aims; in the second round, full-text articles were selected for data extraction. The screening process was conducted by Xin Sheng and verified by Chengwei Liu, Xiaofan Zhang and Fengfei Ding. Any disagreements were resolved through discussion among the authors.

### Inclusion and exclusion criteria

A comprehensive search of studies involving SD experiments was conducted using three databases: PubMed, Scopus and Embase. The behavioural tests employed to assess memory function include the fear conditioning test (FC), Morris water maze test (MWM), novel object recognition test (NOR), novel location recognition test (NLR), passive avoidance test (PAT), plus-maze discriminative avoidance task (PMDAT) and radial arm water maze task (RAWM).^25-30^ Behavioural tests used to evaluate depression-like behaviours comprise the forced swimming test (FST), sucrose preference test (SPT) and tail suspension test (TST).^31-33^ Additionally, the behavioural tests assessing anxiety-like behaviours include the elevated plus maze test (EPM), open field test (OF) and PMDAT.^34-36^ The parameters of each behavioural test are presented in Supplementary Table 2. Included studies should identify SD as the independent variable and behavioural changes as the dependent outcomes. For studies incorporating additional variables beyond SD, such as transgenic manipulation or pharmacological treatments, data from littermates or vehicle controls subjected to SD protocols were also considered. Studies excluded from our analysis included: (i) reviews/systematic reviews; (ii) studies involving other animal species; (iii) those reporting only title/abstract; and (iv) studies with incompatible designs, including those lacking data for calculating standardized mean values and SDs, those without a defined primary outcome or control group, those that did not provide sample sizes and those exhibiting heterogeneity in behavioural experiments.

### Data extraction and processing

Data were primarily extracted directly from written results in the text. If necessary, we inquired about the raw data from the authors via email. Subsequently, we manually measured the histograms using a digital ruler (Image J) to extract the mean and standard deviation values for studies that did not respond to our requests for results. For each experiment, detailed information regarding the SD protocols was compiled, including species information (mouse or rat), duration of SD and sample size (n). In cases where the sample size was reported as a range rather than exact numbers, we used the minimum value of the range as the sample size. The data were presented as the standard error of the mean (SEM), and the standard deviation was calculated using the formula: Standard deviation = SEM ×$\sqrt{n}$.

To investigate the global effect of experimental SD on behavioural tests, we combined data from the same experimental setup across different articles into a single group. The details of the algorithm are provided in the supplementary methods.^37^

### Statistical analysis

#### Meta-analysis

We conducted multiple meta-analyses with four clusters of datasets. Initially, all included studies were aggregated to assess the behavioural outcomes of SD, encompassing all paradigms. For each behavioural test parameter, we integrated the sample sizes and measurements from various studies to derive an overall sample size, mean and standard deviations, as detailed in the Supplementary material. Subsequently, we stratified the datasets into subgroups to examine factors influencing the results. Further meta-analyses were performed within these subgroups to compare REMSD versus SD protocols, short-term versus long-term protocols and species differences between mice and rats.

The analysis encompassed the first three clusters of datasets. Cluster one examines the overall effects of SD (including all paradigms) and REMSD protocols on memory function, as well as depression- and anxiety-like behaviours. Cluster two investigates the impact of deprivation protocols (SD or REMSD) on behavioural outcomes across species, specifically mice or rats. Cluster three evaluates the effects of deprivation protocols (SD or REMSD) on outcomes over varying durations, distinguishing between short-term and long-term deprivation. Protocols exceeding 72 h were categorized as long-term, while those lasting <72 h were classified as short-term.

We subsequently conducted a meta-analysis to identify behavioural tests and their respective parameters with sufficient sensitivity for detecting behavioural alterations following SD. We calculated the standardized mean difference (SMD) for each parameter of the behavioural tests across different studies using the Cohen method (Cluster Four).^38^ Unlike clusters one to three, which utilized combined data on sample size, mean and standard deviation from multiple studies, the analysis in cluster four relied on the extracted data from individual research studies.

We employed a DerSimonian and Laird random effects weighted mean difference meta-analysis to calculate the overall effect size for each dataset cluster.^39^ Data were presented as SMD with 95% confidence intervals (95% CI). Results were deemed significant if the confidence interval did not include zero and the associated Cochran's Q *P*-value was <0.05. All calculations were conducted using Stata 17 software (STATA Corporation, College Station, TX, USA). The effect size was further analysed using ANOVA in IBM SPSS version 25.0, with a *P*-value below 0.05 considered indicative of statistical significance.

#### Risk of bias and quality assessment

**Figure 1:**
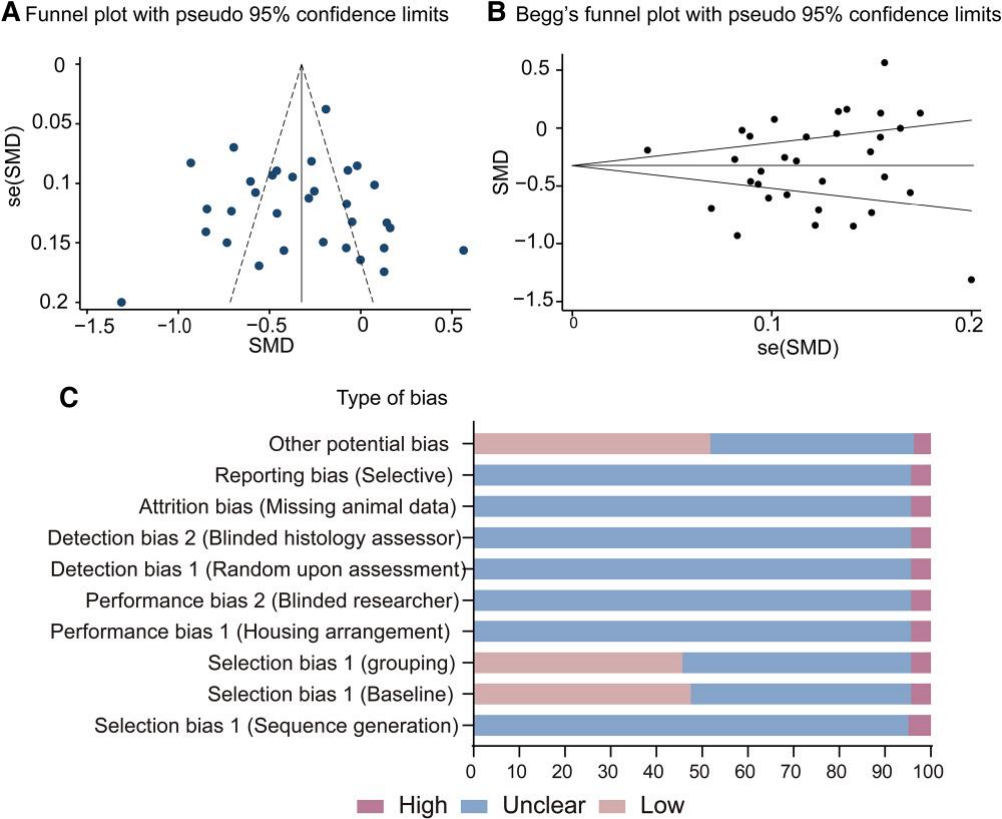
**Publication bias with global effects of sleep deprivation.** (**A**) The funnel plot was constructed to illustrate the relationship between the study's precision [standard error of the standardized mean difference, S.E. (SMD)] and effect sizes (SMD) (*n* = 32). (**B**) Begg's funnel plot was utilized to assess publication bias concerning the global effects of sleep deprivation. The horizontal line in the funnel plot represents the random-effects summary estimate, while the sloping lines indicate the expected 95% CI for the standard error (*n* = 32). (**C**) A summary of outcomes derived from the SYRCLE's Risk of Bias (RoB) assessment tool is presented (*n* = 164). Each dot in Fig. 1A and B represents the combined data from the analysis of the global effects of sleep deprivation. *n*, sample size; SMD, standardized mean difference.

The selection bias was evaluated using SYRCLE's Risk of Bias (RoB) tool.^40^ To mitigate the influence of sample size in the subsequent subgroup meta-analysis, we generated a funnel plot based on the combined data from the global effects of SD analysis to assess publication bias. The publication bias of all selected articles was evaluated through visual analysis of the funnel plot (meta funnel plot, STATA Corporation, College Station, TX, USA).^41^ This funnel plot was constructed using the study's precision (standard error) and effect size. We employed Egger's regression test to assess potential publication bias.^42^ Visual inspection of the funnel plot revealed that effect sizes were evenly distributed across the top of the funnel, indicating no significant publication bias in the meta-analyses (Fig. 1A). A quantitative assessment using Begg's test further confirmed the absence of publication bias (*P* = 0.783, Fig. 1B). The shortlisted studies exhibited either low or unclear risk of bias in most assessment categories, according to SYRCLE's RoB tool (Fig. 1C). Given the absence of publication bias in the meta-analysis of global effects, we disregarded publication bias in the subsequent subgroup meta-analysis.

## Results

**Figure 2:**
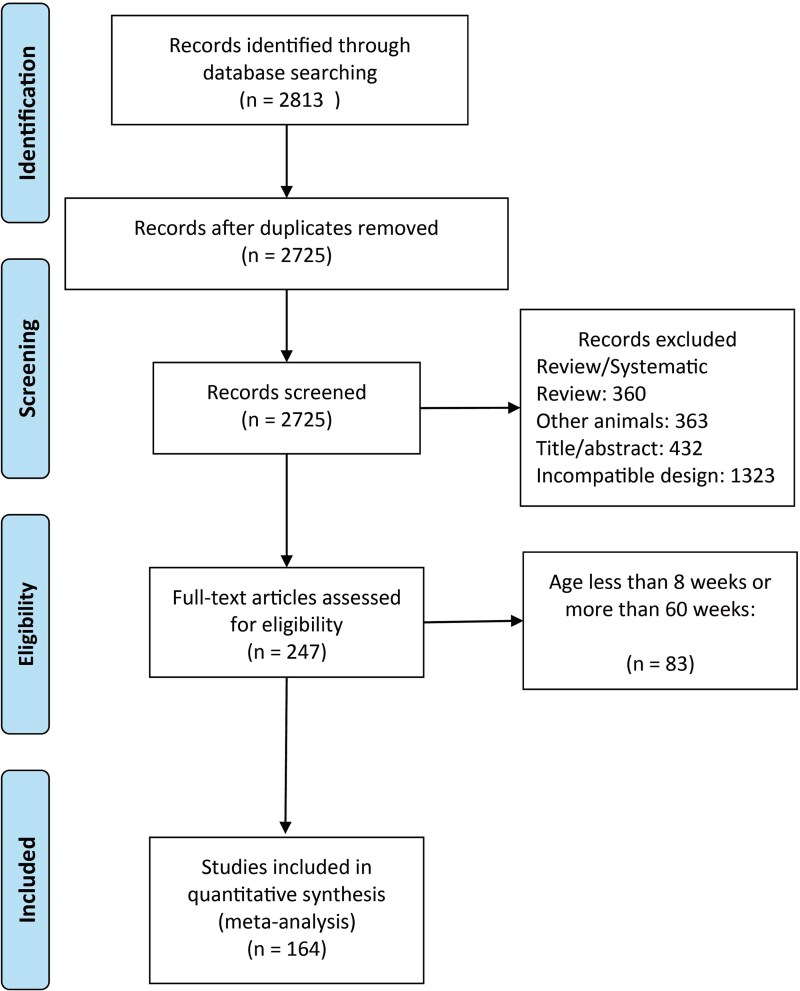
**Flowchart of the articles selection process.**  *n*, sample size.

The study selection process is illustrated in Fig. 2. A total of 2813 articles were screened based on their abstracts, with full-text articles consulted as necessary. Following the initial screening, 2725 original studies underwent a comprehensive evaluation, resulting in the acquisition of full texts for 247 articles for data extraction. Articles featuring rats or mice younger than 8 weeks or older than 60 weeks were excluded, leading to the inclusion of 164 articles in the final meta-analysis. Among these, 17 articles focused exclusively on females, 134 on males, 9 on both genders and 4 did not specify gender. Some articles reported multiple behavioural assessments and SD paradigms, yielding a total of 371 indices for the current meta-analysis. These indices were derived from 164 articles (REMSD: *n* = 104, TSD: *n* = 44, Sleep Restriction: *n* = 5, Sleep Fragmentation: *n* = 6, REMSD and TSD: *n* = 2, REMSD and Sleep Restriction: *n* = 2, REMSD and Sleep Fragmentation: *n* = 1; Supplementary Table 2) and encompassed 306 experiments. Of the articles reviewed, 62 utilized mice and 102 utilized rats, with the following mouse strains represented: BALB/c (*n* = 3), C57BL/6 (*n* = 37), CD-1 (*n* = 1), DBA/2J (*n* = 1), ICR (*n* = 2), Swiss (*n* = 17) and 1 article did not disclose strain information. For rats, the strains included Long Evans (*n* = 4), Sprague Dawley (*n* = 24), Wistar (*n* = 72) and two articles did not disclose strain information.

Among these, 109 articles and 207 experiments specifically investigated REMSD. A comprehensive description of these articles and experiments, including the details of the rat and mouse strains, duration of the studies and behavioural categories, is presented in Table 1. Additionally, the specifics of the variables included in the meta-analysis can be found in Supplementary Table 2.

**Table 1:** The number of articles or experiments for subgroups

	SD		REMSD	
	Articles (*n*)	Exp *(N)*	Articles (*N*)	Exp (*N*)
**Total**	164	306	109	207
**Species**				
Mice	62	130	30	71
Rats	102	176	79	136
**Duration**				
Long-term	59	110	39	84
Short-term	113	196	70	123
**Memory function**				
FC	24	28	8	9
MWM	53	56	36	40
NOR	28	35	17	22
NLR	9	11	7	7
PAT	14	23	12	18
PMDAT	4	6	2	3
RAWM	16	35	17	35
**Depressive-like behaviour**				
FST	22	35	12	20
SPT	8	14	6	12
TST	7	8	3	3
**Anxiety-like behaviour**				
EPM	20	20	12	15
OF	24	31	16	22
PMDAT	3	4	1	1

A single article may have employed more than one behavioural assessment. EPM, elevated plus maze test; FC, fear conditioning test; FST, forced swim test; MWM, Morris water maze test; n, sample size; NLR, novel location recognition test; NOR, novel object recognition test; OF, open field test; PAT, passive avoidance test; PMDAT, plus-maze discriminative avoidance task; RAWM, radial arm water maze test; REMSD, rapid eye movement sleep deprivation; SD, sleep deprivation; SPT, sucrose preference test; TST, tail suspend test.

### Both SD and REMSD induced memory disruption and depression-like behaviours; only SD induced anxiety-like behaviour

**Figure 3:**
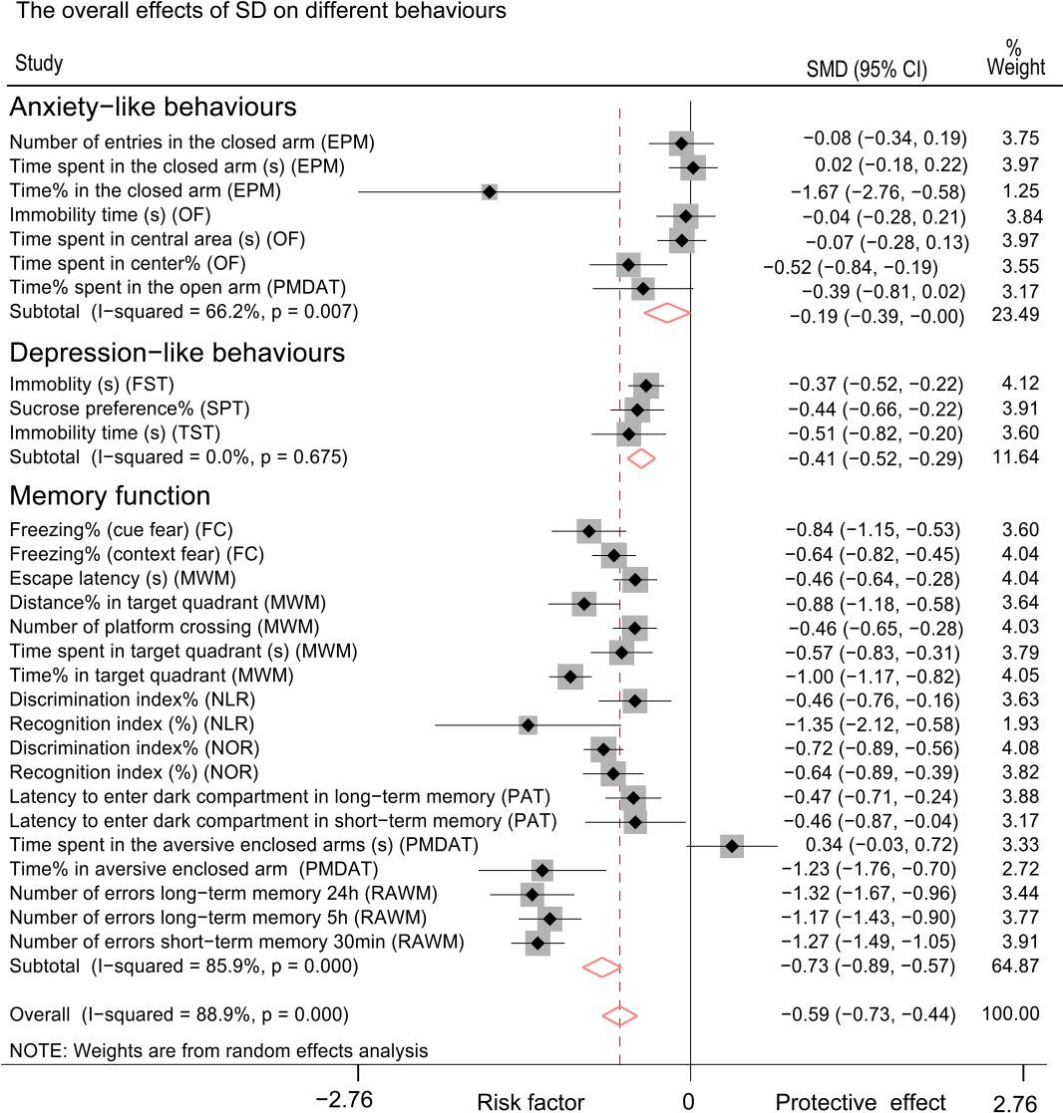
**Forest plot for the effects of SD on anxiogenesis, depression and memory function loss, respectively.** Memory function: control group sample size (Con-n) = 2514, Experimental group sample size (Exp-n) = 2533. Depression-like behaviours: control group sample size (Con-n) = 588, experimental group sample size (Exp-n) = 596. Anxiety-like behaviours: Control group sample size (Con-n) = 735, Experimental group sample size (Exp-n) = 719. The black square and horizontal line represent each study's odds ratio and 95% CI, respectively, with a point estimate.^43^ The black diamond indicates the effect size of each study. The summary effect for each subgroup and overall is visualized as the diamond at the bottom of the plot.^44^ CI, confidence interval; EPM, elevated plus maze test; FC, fear conditioning test; FST, forced swim test; MWM, Morris water maze test; n, sample size; NLR, novel location recognition test; NOR, novel object recognition test; OF, open field test; PAT, passive avoidance test; PMDAT, plus-maze discriminative avoidance task; RAWM, radial arm water maze test; SD, sleep deprivation; SMD, standardized mean difference; SPT, sucrose preference test; TST, tail suspend test.

**Figure 4:**
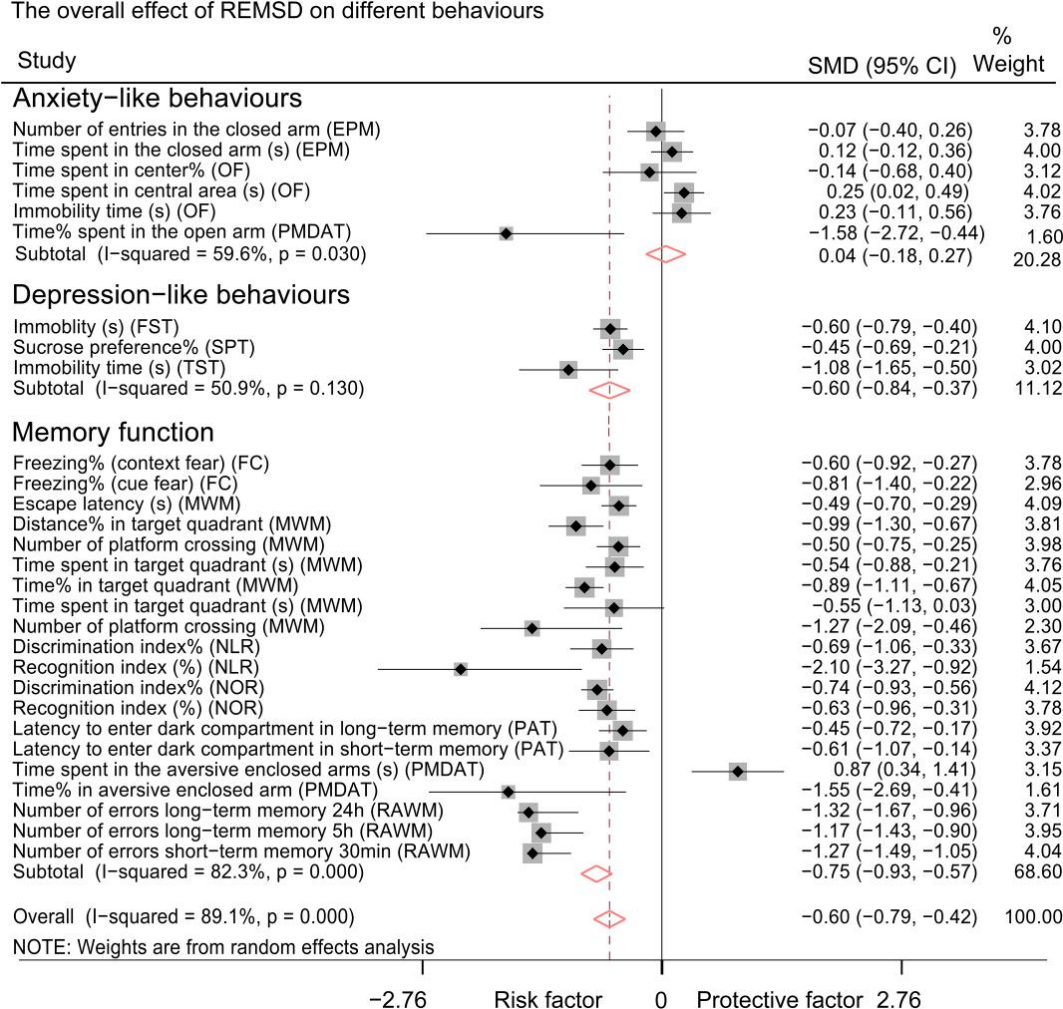
**Forest plot for the effects of REMSD on anxiogenesis, depression and memory function loss, respectively.** Memory function: Control group sample size (Con-n) = 1745, Experimental group sample size (Exp-n) = 1768. Depression-like behaviours: Control group sample size (Con-n) = 371, Experimental group sample size (Exp-n) = 372. Anxiety-like behaviours: Control group sample size (Con-n) = 438, Experimental group sample size (Exp-n) = 446. The meanings of the black square, horizontal line and black and diamonds in the figure are consistent with those in Fig. 3. CI, confidence interval; EPM, elevated plus maze test; FC, fear conditioning test; FST, forced swim test; MWM, Morris water maze test; n, sample size; NLR, novel location recognition test; NOR, novel object recognition test; OF, open field test; PAT, passive avoidance test; PMDAT, plus-maze discriminative avoidance task; RAWM, radial arm water maze test; REMSD, rapid eye movement sleep deprivation; SMD, standardized mean difference; SPT, sucrose preference test; TST, tail suspend test.

Through a comprehensive meta-analysis of all parameters extracted from behavioural tests, we found that both SD (encompassing all SD paradigms) and REMSD significantly disrupted behaviours across all selected experiments involving rats and mice [*P* < 0.001, SMD (95% CI): −0.59 (−0.73, −0.44)for SD; *P* < 0.001, SMD (95% CI): −0.60 (−0.79, −0.42) for REMSD]. The subgroup meta-analysis revealed that SD was associated with the induction of anxiety-like behaviours [*P* = 0.049, SMD (95% CI): −0.19 (−0.39, −0.00)], depression-like behaviours [*P* < 0.001, SMD (95% CI): −0.41 (−0.52, −0.29)] and memory loss [*P* < 0.001, SMD (95% CI): −0.73 (−0.89, −0.57), Fig. 3]. In contrast, REMSD was found to induce only depression-like behaviours [*P* < 0.001, SMD (95% CI): −0.60 (−0.79, −0.42)] and memory loss [*P* < 0.001, SMD (95% CI): −0.75 (−0.93, −0.57)] but not anxiety-like behaviours [*P* = 0.705, SMD (95% CI): 0.04 (−0.18, 0.27)] in rats and mice (Fig. 4).

### Only SD-induced anxiety-like behaviour in rats

**Figure 5:**
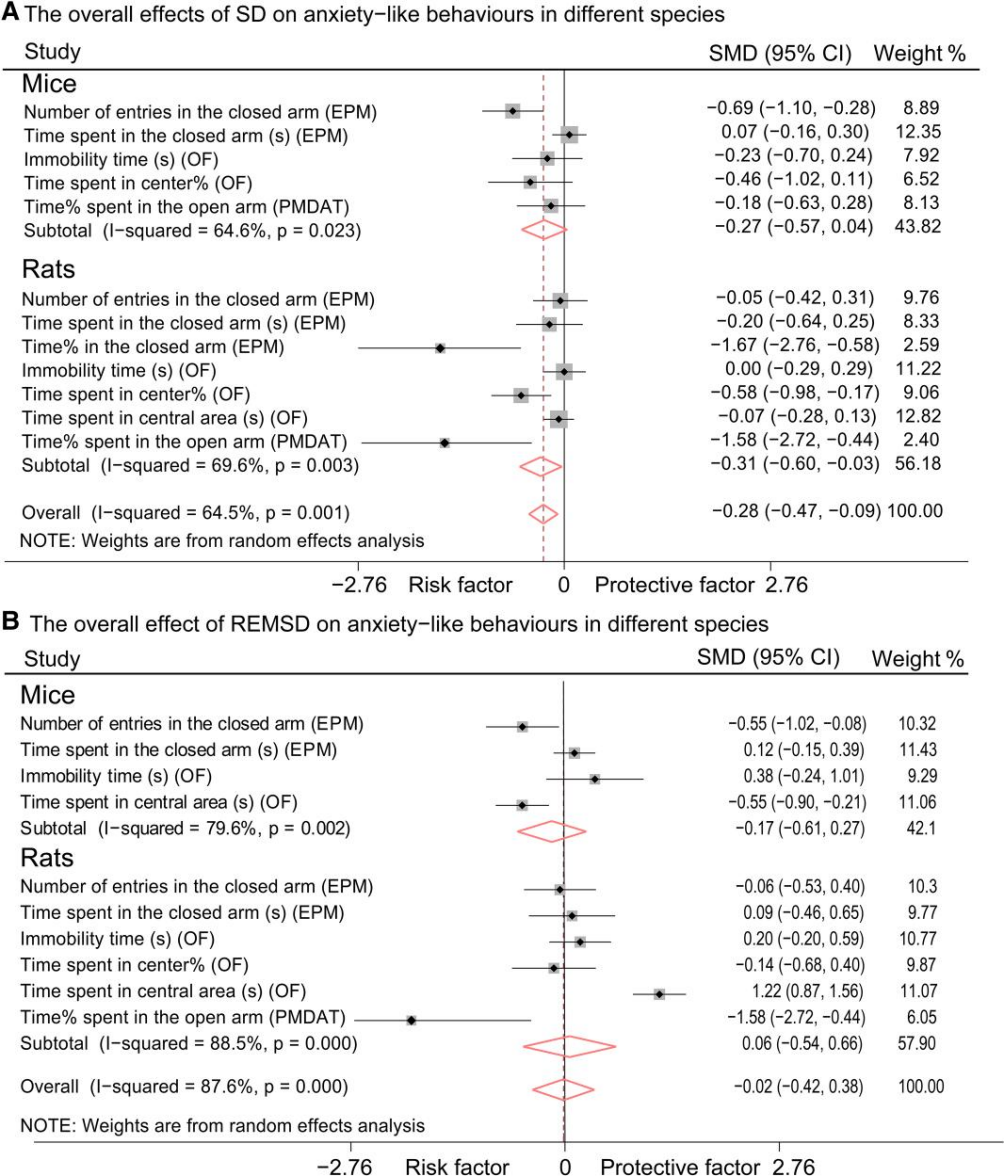
**Forest plots the effects of SD (A) and REMSD (B) on anxiety-like behaviours between mice and rats.** (**A**) The overall effects of SD on anxiety-like behaviours in different species. (**B**) The overall effect of REMSD on anxiety-like behaviours in different species. Anxiety-like behaviours: SD: Mice: Control group sample size (Con-n) = 307, Experimental group sample size (Exp-n) = 283; Rats: Control group sample size (Con-n) = 428, Experimental group sample size (Exp-n) = 436. REMSD: Mice: Control group sample size (Con-n) = 224, Experimental group sample size (Exp-n) = 233; Rats: Control group sample size (Con-n) = 214, Experimental group sample size (Exp-n) = 213. The meanings of the black square, horizontal line and black and diamonds in the figure are consistent with those in Fig. 3. CI, confidence interval; EPM, elevated plus maze test; n, sample size; OF, open field test; PMDAT, plus-maze discriminative avoidance task; REMSD, rapid eye movement sleep deprivation; SD, sleep deprivation; SMD, standardized mean difference.

We conducted a meta-analysis based on studies involving different species to compare the effects of SD on anxiety-like behaviours between rats and mice. Our findings indicated that the SD protocols led to the emergence of anxiety-like behaviours in rats [*P* = 0.030, SMD (95% CI): −0.31 (−0.60, −0.03)] but not in mice [*P* = 0.088, SMD (95% CI): −0.27 (−0.57, 0.04), Fig. 5A]. In contrast, neither mice nor rats exhibited anxiolytic-like behaviours following REMSD [*P* = 0.453, SMD (95% CI): −0.17 (−0.61, 0.27) for mice; *P* = 0.844, SMD (95% CI): 0.06 (−0.54, 0.66) for rats, Fig. 5B]. Additionally, both species displayed depressive-like behaviours and memory loss following treatments with either SD or REMSD (Table 2).

**Table 2:** The pooled effects of SD and REMSD by different rodent species and deprivation protocol durations

Categories	SMD	95%CI	Random effects *P*-value
**Rodent species**			
**Memory function**			
SD on mice	−0.56	−0.82, −0.30	0.000***
SD on rats	−0.84	1.02, −0.67	0.000^***^
REMSD on mice	−0.56	−0.90, −0.22	0.001^***^
REMSD on rats	−0.86	−1.08, −0.65	0.000***
**Depression-like behaviours**			
SD on mice	−0.36	−0.49, −0.23	0.000***
SD on rats	−0.93	−1.59, −0.27	0.005**
REMSD on mice	−1.23	−2.18, −0.28	0.011*
REMSD on rats	−0.93	−1.60, −0.27	0.006**
**Anxiety-like behaviours**			
SD on mice	−0.27	−0.57, 0.04	0.088
SD on rats	−0.39	−0.70, −0.09	0.012*
REMSD on mice	−0.17	−0.61, 0.27	0.453
REMSD on rats	0.06	−0.54, 0.66	0.844
**Protocol duration**			
**Memory function**			
Long-term SD	−0.99	−1.20, −0.78	0.000***
Short-term SD	−0.66	−0.83, −0.48	0.000***
Long-term REMSD	−1.10	−1.30, −0.89	0.000***
Short-term REMSD	−0.67	−0.89, −0.44	0.009**
**Depression-like behaviours**			
Long-term SD	−0.25	−0.47, −0.03	0.003**
Short-term SD	−1.31	−2.19, −0.44	0.026*
Long-term REMSD	−1.36	−2.07, −0.65	0.000***
Short-term REMSD	−0.61	−0.94, −0.28	0.000***
**Anxiety-like behaviours**			
Long-term SD	−0.04	−0.42, 0.34	0.829
Short-term SD	−0.24	−0.47, 0.00	0.054
Long-term REMSD	0.03	−0.36, 0.43	0.548
Short-term REMSD	0.12	0.27, 0.50	0.870

REMSD, rapid eye movement sleep deprivation; SD, sleep deprivation; SMD, the standardized mean difference; ∗*P* ≤ 0.05, ∗∗*P* ≤ 0.01, ∗∗∗*P* ≤ 0.001.

### Short-term SD-induced anxiogenesis

**Figure 6:**
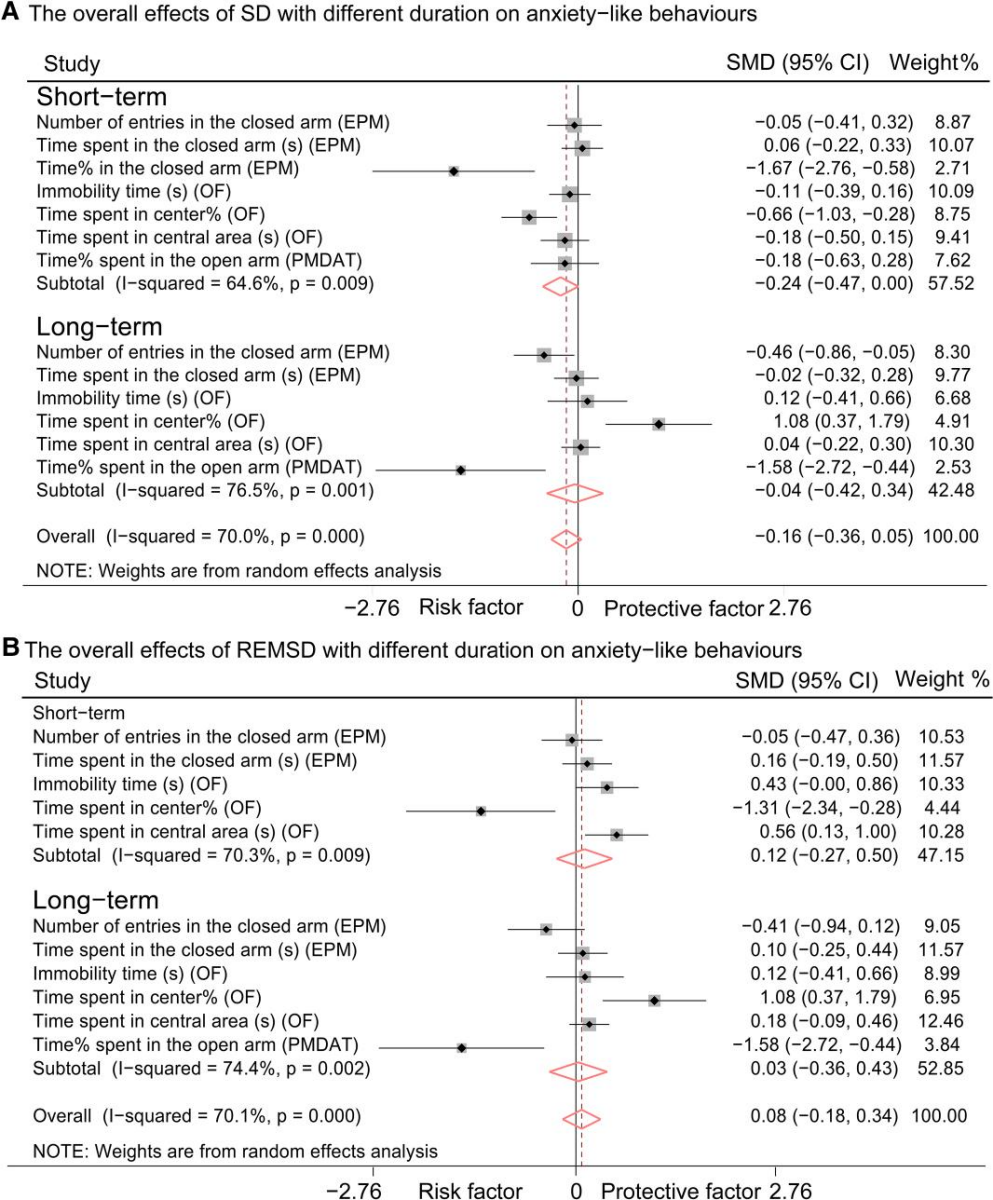
**Forest plot of long-term and short-term SD (A) and REMSD (B) on anxiety-like behaviours.** (**A**) The overall effects of SD with different duration on anxiety-like behaviours. (**B**) The overall effects of REMSD with different duration on anxiety-like behaviours. SD: Long-term: Control group sample size (Con-n) = 296, Experimental group sample size (Exp-n) = 292; Short-term: Control group sample size (Con-n) = 439, Experimental group sample size (Exp-n) = 427. REMSD: Long-term: Control group sample size (Con-n) = 246, Experimental group sample size (Exp-n) = 254; Short-term: Control group sample size (Con-n) = 192, Experimental group sample size (Exp-n) = 192. The meanings of the black square, horizontal line and black and diamonds in the figure are consistent with those in Fig. 3. CI, confidence interval; EPM, elevated plus maze test; n, sample size; OF, open field test; PMDAT, plus-maze discriminative avoidance task; REMSD, rapid eye movement sleep deprivation; SD, sleep deprivation; SMD, standardized mean difference.

Similarly, the analysis of pooled samples revealed that both long-term and short-term SD and REMSD induced depression-like behaviours and memory deficits in rats or mice (Table 2). Notably, only short-term SD was associated with increased anxiety levels in these animals, as indicated by a trend towards significance [short-term SD: *P* = 0.054, SMD (95% CI): −0.24 (−0.47, 0.00); long-term SD: *P* = 0.829, SMD (95% CI): −0.04 (−0.42, 0.34), as illustrated in Fig. 6A]. In contrast, neither short-term nor long-term REMSD produced significant alterations in anxiety-like behaviours [short-term REMSD: *P* = 0.548, SMD (95% CI): 0.12 (−0.27, 0.50); long-term REMSD: *P* = 0.870, SMD (95% CI): 0.03 (−0.36, 0.43), as depicted in Fig. 6B].

### The best parameters to detect memory disruption, depression and anxiety-like behaviours due to SD and REMSD

In the meta-analysis of Cluster four, we calculated the SMD for the individual parameters of the behavioural tests to identify those with adequate sensitivity for detection. The FC, MWM, NOR, NLR, PAT and RAWM are effective in detecting cognitive decline associated with both SD and REMSD through observable behavioural changes related to memory function. For assessing depression-like behaviours, the FST, SPT and TST proved to be reliable indicators of depressive behaviours following SD or REMSD. In terms of evaluating anxiety-like behaviours, the EPM successfully identified anxiety-like responses in both SD and REMSD conditions. Conversely, the PMDAT was limited to detecting changes specifically related to REMSD. Notably, the OF failed to identify anxiety-like behaviours in either SD or REMSD conditions (Supplementary Table 3).

**Figure 7:**
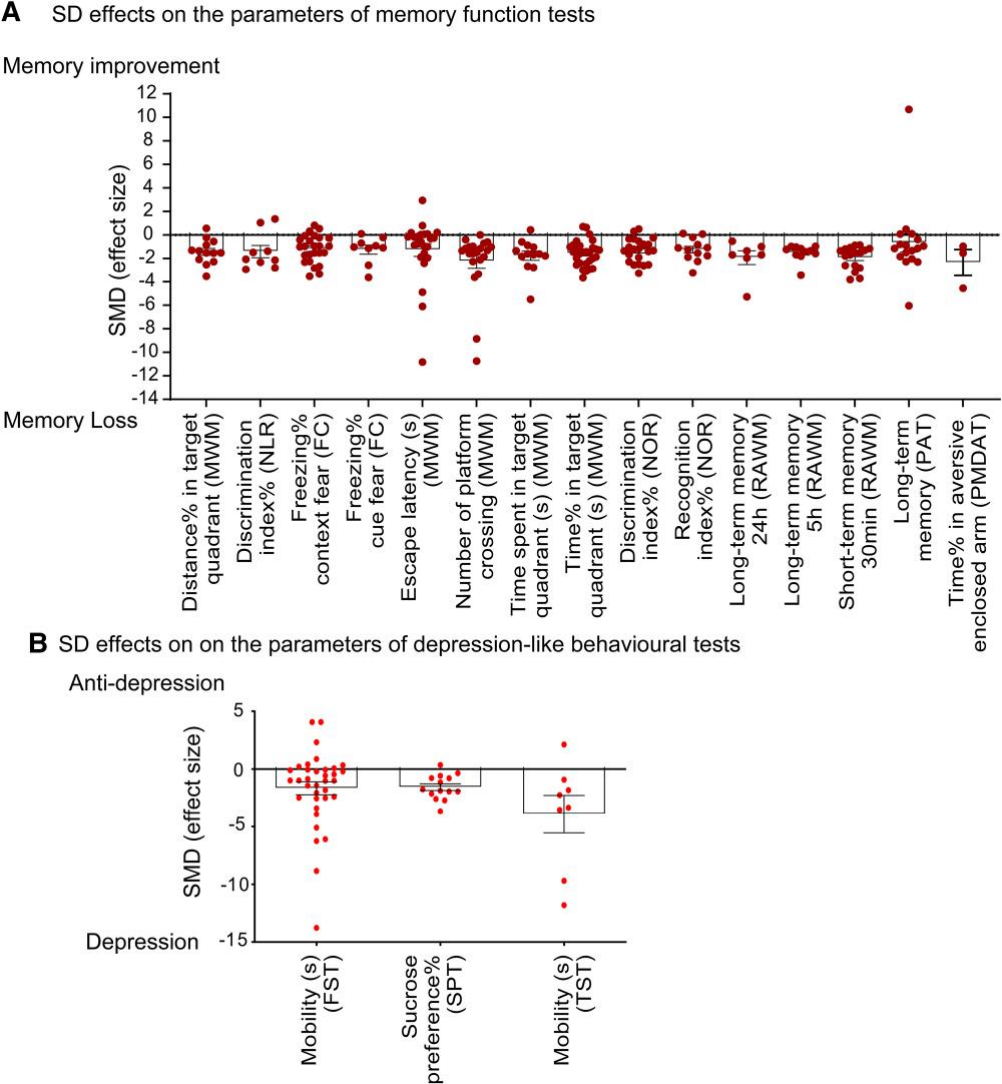
**The effect size of the parameters to evaluate memory function and depressive-like behaviours after sleep deprivation.** The differences were assessed using one-way ANOVA. The sensitivity of the parameters revealed no significant differences in (**A**) memory function tests [*F*(14, 217) = 0.809, *P* = 0.659] and (**B**) depressive-like behaviour tests [*F*(2, 54) = 2.587, *P* = 0.085]. The effect sizes of the selected parameters that demonstrated statistical significance were derived from the meta-analysis of cluster four (Supplementary Table 3). These parameters were obtained from behavioural tests evaluating memory function and depressive-like behaviours, including FC, FST, MWM, NLR, NOR, PAT, RAWM, TST and SPT. (**A**) Memory Function Tests: Freezing% context fear (FC), *n* = 25; Freezing% cue fear (FC), *n* = 9; Escape Latency(s) (MWM), *n* = 24; Distance% in target quadrant (MWM), *n* = 13; Number of platform crossing (MWM), *n* = 21; Time spent in target quadrant(s) (MWM), *n* = 13; Time% in target quadrant (MWM), *n* = 29; Discrimination index% (NLR), *n* = 9; Discrimination index% (NOR), *n* = 22; Recognition index% (NOR), *n* = 12; Long-term memory (PAT), *n* = 17; Time% in aversive enclosed arm (PMDAT), *n* = 3; Long-term memory 24 h (RAWM), *n* = 7; Long-term memory 5 h (RAWM), *n* = 11; Short-term memory 30 min (RAWM), *n* = 17. (**B**) Depressive-like Behaviour Tests: Immobility (S) (FST), *n* = 35; Sucrose preference% (SPT), *n* = 14; immobility (S) (TST), *N* = 8. Each dot represents the effect size generated from one experiment. Data are presented as mean ± SEM. FC, fear conditioning; FST, forced swim test; MWM, Morris water maze; n, sample size; NLR, novel location recognition; NOR, novel object recognition; PAT, passive avoidance test; RAWM, radial arm water maze; SD, sleep deprivation; TST, tail suspend test; SPT, the sucrose preference test.

The parameters with a *P*-value <0.05 in the meta-analysis (Supplementary Table 3) were plotted alongside their SMD, which reflect the sensitivity of detection regarding the behavioural outcomes of either SD or REMSD. Given that SD and REMSD exhibited similar effects across different species and experimental durations on memory function and depression-like behaviours, we focused solely on parameters derived from SD paradigms for these behavioural tests. The sensitivity of the parameters did not show significant differences among the memory function tests [*F*(14, 217) = 0.809, *P* = 0.659]. Notably, the number of platform crossings and the time spent (both in percentage and seconds) in the target quadrant during the MWM test demonstrated the highest average SMD among the parameters for detecting memory impairments induced by SD and REMSD (Fig. 7A). In terms of depression-like behaviours, the immobility score from the TST exhibited the most robust performance in detecting these behaviours in both SD and REMSD conditions (Fig. 7B). However, the sensitivity of the parameters did not reveal significant differences among the tests for depression-like behaviours [*F*(2, 54) = 2.587, *P* = 0.085].

The parameter representing the number of entries in the closed arm of the EPM exhibited comparable efficacy in detecting anxiety-like behaviours under both SD and REMSD conditions [one-way ANOVA: *F*(2, 27) = 0.111, *P* = 0.896; Supplementary Fig. 1]. The anxiety-like effects observed were sufficiently robust to indicate that they were independent of rodent species, as demonstrated by a two-way ANOVA revealing no significant effects for parameters [*F*(2, 31) = 0.513, *P* = 0.726) or species (*F*(1, 31) = 0.685, *P* = 0.414]. Furthermore, the duration of SD did not significantly influence anxiety-like behaviours, as indicated by the two-way ANOVA results for parameters [*F*(2, 31) = 0.040, *P* = 0.961) and duration (*F*(1, 31) = 0.003, *P* = 0.957; Supplementary Fig. 1].

However, the immobility score was found to be above zero in rats subjected to REMSD (Supplementary Fig. 2). In contrast to the findings observed in cluster two, REMSD appears to exert a mild anxiolytic effect in rats, with scores nearly above zero, while simultaneously eliciting an anxiogenic effect in mice (Supplementary Fig. 2A). Furthermore, this parameter demonstrated slightly enhanced sensitivity in detecting anxiety-like behaviours during short-term REMSD compared with long-term REMSD (Supplementary Fig. 2B). These findings underscore the importance of focusing on these parameters when evaluating the behavioural outcomes associated with SD.

## Discussion

Through meta-analysis, we synthesized the experimental findings regarding the behavioural outcomes following SD interventions in both rats or mice. Our analysis indicates that SD protocols consistently lead to deficits in memory function, irrespective of the specific SD paradigms, modelling durations, or species involved (Figs 3 and 4 and Table 2). When comparing effect sizes, the MWM test, which assesses spatial memory function in rodents,^45^ emerged as the most sensitive behavioural assessment for memory function among the tests evaluated (Fig. 7). From an ethical standpoint, intensive SD interventions are typically limited to healthy young individuals for a maximum duration of a few days.^46-48^ Research has shown that a five-day sleep restriction impairs spatial working memory function without affecting verbal working memory or declarative memory in humans.^49^ Additionally, one study implementing mild sleep restriction over six weeks reported subsequent impairments in spatial orientation and vigilant attention.^50^ Our findings align with these observations in humans, highlighting the strong association between sleep loss, cognitive deficits and neurodegeneration.^51,52^ Both rodent and human studies have demonstrated that SD negatively impacts brain interstitial waste clearance.^53-55^ Therefore, the conclusions drawn from our research support the rationale for utilizing rat and mouse models to elucidate the mechanisms underlying sleep loss and to serve as valuable disease models for therapeutic development.

It was surprising to discover that both short-term and long-term SD or REMSD could induce depression-like behaviours in healthy rats and mice (Table 2). Traditional animal models of depression, such as chronic unpredictable mild stress and social isolation, typically require 1 to 2 months for effective modelling.^56,57^ This finding aligns with clinical observations indicating that chronic sleep loss can lead to depression in healthy individuals.^58,59^ Interestingly, some studies have reported that acute SD may produce an antidepressant effect in depressed patients,^60-62^ although these individuals often relapse into depression following a recovery night.^63^ In our analysis, we found that the immobility time measured by the TST exhibited the best detection performance compared with the sucrose preference percentage in the SPT and the immobility time in the TST (Fig. 7B). The TST is a commonly used assessment for screening potential antidepressant drugs.^33^ Additionally, SD protocols also yielded positive findings in both the FST and SPT. We speculate that the results of these assessments may be influenced by reduced locomotion due to sleep debt, particularly following acute SD. Therefore, caution is warranted when correlating depression-like behaviours in rodents with actual ‘depression’ following acute SD, as these models lack subjective emotional assessments.^64^ A recent study reported similar depression-like behavioural outcomes in the FST, SPT and TST following SD.^65^ While they reviewed the literature concerning REMSD, SD and various rodent species, they did not compare factors such as mice versus rats or REMSD versus SD, which would highlight the differential effects of various SD protocols.^65^

In comparison to the deficits in memory function and the presence of depression-like behaviours, the anxiety-like behaviours resulting from SD or REMSD were less consistent (Fig. 5). SD was found to induce anxiety-like behaviours in rats (Fig. 5A and Table 2), particularly with short-term SD, which demonstrated an overall anxiogenic effect (Fig. 6A; Table 2). Conversely, REMSD did not yield significant results across the overall battery of anxiety-like behaviours (Figs 5B and 6B;  Table 2), a finding that aligns with previous studies conducted on both mice and rats.^13^ Research on SD-induced anxiety-like behaviour in animal models has not mirrored the anxiety observed in humans following SD.^4^ Consequently, experimental SD has been deemed to lack translational value in this context.^66-68^ It is important to note that the mood-elevating effects of SD reported in some studies are not observed in healthy human volunteers.^59^ The subjects in our study were all wild-type rats and mice, receiving no additional treatments aside from the SD intervention; therefore, data on animal models of mood disorders remain limited. Furthermore, the response criteria used in clinical trials often rely on categorical judgments (e.g. ‘was helpful’ versus. ‘was not helpful’), which are based on personal subjective assessments, or they may refer to minimal differences in absolute scores or percentages from questionnaires.^69,70^ In contrast, response criteria in rodent studies primarily focus on behavioural phenotypes.^71^ This discrepancy in rating practices may contribute to the heterogeneity observed between clinical trial outcomes and experimental animal study results. Additionally, diverse behavioural outcomes reported in both rodents and humans, such as ‘hyperactivity,’ impulsivity, aggression and ‘manic’ states, could be associated with anxiety.^72-77^ REMSD has been shown to alter levels of various neurotransmitters, including dopamine, serotonin, norepinephrine and γ-aminobutyric acid, all of which are linked to adverse behavioural changes and other health consequences, including mania and various psychiatric disorders.^78^

Pires *et al*.^4^ summarized the anxiety-like behaviours resulting from SD, and their findings regarding the EPM, OF and PMDAT were consistent with our current analysis. However, contrary to the anxiolytic effects of REMSD reported in Pires’s study, we did not observe any significant impacts of REMSD on anxiety-like behaviours. It is worth noting that TSD may elicit a more pronounced stress response, with anxiogenic effects predominantly associated with TSD.^79^ In our study, 46 articles utilized TSD (Supplementary Table 2), while only 11 articles in Pires’s review involved TSD. This disparity suggests that TSD may play a significant role in the anxiogenesis observed in our findings. Moreover, our study incorporated multiple variables within each behavioural test paradigm, whereas Pires’s study restricted each analysis to a single variable category, potentially leading to a reduced sample size that could bias their results. Both our study and Pires *et al*. identified inconsistencies in anxiety-like behaviours resulting from SD. Specifically, we found that SD protocols induced anxiety-like behaviours in rats but not in mice [*P* = 0.030, SMD (95% CI): −0.31 (−0.60, −0.03) for rats; *P* = 0.088, SMD (95% CI): −0.27 (−0.57, 0.04) for mice; Fig. 5A]. This conclusion was based on the overall effects observed across all anxiety-like behavioural tests (EPM, PMDAT and OF). Notably, only the EPM effectively detected anxiety-like behaviours in the context of SD (Supplementary Table 3). We speculate that the limitations of certain studies may hinder the achievement of statistically significant results. Nevertheless, the findings in mice approached significance, with the upper limit of the 95% CI being 0.04, which is nearly zero. Consistently, parameters utilized in the EPM test indicated an anxiogenic effect in mice (Supplementary Fig. 2A). The non-significant result for mice suggests that some behavioural tests may lack sufficient sensitivity to assess anxiety-like behaviours in mice subjected to SD manipulation. Our study offers insights that may help clarify the inconsistencies observed in behavioural outcomes across different studies.

Similar to depression-like behaviours, evaluating anxiety-like behaviours in animal models is even more complex than in humans, primarily due to numerous confounding factors that can influence behavioural assessments, such as genetic background, sex and environmental conditions.^80^ In our review, we identified 14 articles that focused on anxiety-like behaviours (non-REMSD: *n* = 9, REMSD: *n* = 5) utilizing C57BL/6 mice, while seven articles (non-REMSD: *n* = 1, REMSD: *n* = 6) employed Swiss mice. Although C57BL/6 mice are generally recognized as being less active and exhibiting higher anxiety levels compared with Swiss mice, we are unable to draw definitive conclusions regarding the impact of mouse strains on the results due to the disproportionate representation of REMSD manipulations (Supplementary Table 2). Moreover, the timing of behavioural experiments was not specified in the majority of the studies we reviewed, which could represent a potential source of variability in the results. Despite these limitations, our analysis still indicated that species and the duration of SD might also act as confounding factors (Figs 5 and 6). Additionally, it is important to note that SD protocols involving consistent rotating bars or platforms may induce stress in both rats and mice, further complicating the interpretation of anxiety-like behaviours.^81-83^

In contrast, other SD protocols, such as manual gentle handling, tend to be less stressful.^84^ Therefore, SD can serve as a significant source of stress, particularly concerning the duration and intensity of the manipulation. It is nearly impossible to avoid inducing stress, especially during short-term SD interventions. Consequently, we may interpret the anxiety-like behaviours observed in this study as a result of the interplay between short-term SD and stress. Furthermore, animal models used in anxiety research often incorporate behavioural procedures designed to elicit a cognitive or affective state, such as learned helplessness.^85^ It is important to note that learned helplessness may manifest as symptomatic behaviour in rodents, particularly under the long-term effects observed in anxiety-like behavioural studies. Additionally, the concept of sleep debt, as previously discussed, should also be factored into the interpretation of anxiety-like behavioural test results following SD protocols. Both the EPM and OF tests can be influenced by changes in locomotor capabilities, which may introduce bias into the results.^35,86^ The relationship between sleep and anxiety is complex, making it challenging to draw clear conclusions in rat and mouse models.

### Limitations

This meta-analysis concentrated on animal behavioural studies, yet many critical details regarding randomization, blinding, housing conditions and timing of outcome assessments were frequently unreported. Additionally, the studies exhibited varying methodological quality across different laboratories, which significantly impacts the overall quality of the findings. Our analysis included a minimum of three articles per comparison; however, sample size calculations are often absent in the animal literature, leading to the likelihood that many studies are underpowered. Consequently, potential selection bias was unavoidable. The research protocols for behavioural assessments have not been fully standardized, resulting in considerable heterogeneity in the pooled estimates. Furthermore, this meta-analysis did not account for the effects of gender on SD in rats and mice. Previous investigations have explored gender differences in stress responses to SD, revealing that behaviours are closely linked to sex hormone levels.^87^ However, the sample sizes of the included articles (16 focusing on females and nine on both genders, as shown in Supplementary Table 2) were insufficient (15.24%, 25/164) to adequately evaluate the impact of gender across various SD paradigms. Therefore, we opted for a mixed-gender approach in the current meta-analysis. Similarly, due to sample size limitations (as noted in Supplementary Table 4), we were unable to conduct a comprehensive meta-analysis encompassing total SD, sleep restriction and sleep fragmentation. To confirm our findings, large-scale prospective studies examining the effects of SD are still necessary in the future.

## Conclusion

In conclusion, we assert that cognitive impairment induced by SD represents a stable phenotype warranting further mechanistic exploration. The depression- and anxiety-like behaviours resulting from SD are crucial for understanding the neurobiological processes underlying sleep, the pathogenesis of emotional disorders and their comorbidity with sleep disturbances. This study also offers valuable insights for selecting appropriate experimental models to evaluate the efficacy of therapeutics aimed at treating insomnia and its associated complications.

## Supplementary Material

fcaf309_Supplementary_Data

## Data Availability

The data supporting the findings of this study are available within the article and its supplementary materials. Any additional data or materials can be requested from the corresponding author. All calculations were conducted using Stata 17 software, following the guidelines outlined in the Stata meta-analysis reference manual (STATA Corporation, College Station, TX). For further details, please refer to the following link: https://www.stata.com/manuals17/meta.pdf.
