# Supplementary material for: Sleep deprivation affects memory function, depression and anxiety-like behaviours in rats and mice: a systematic review and meta-analysis
Source: Brain Commun. 2025 Aug 28;7(5):fcaf309. doi: 10.1093/braincomms/fcaf309 (PMC12455040; doi:10.1093/braincomms/fcaf309)
Supplement: fcaf309_Supplementary_Data [file fcaf309_supplementary_data.doc]

**Methods:**

The algorithm's details regarding the combined data for assessing the global effect of experimental sleep deprivation on behavioural tests are delineated as follows:^1^

For each group:

Σx = mean * n;

Σx2 = SD2(n-1)+((Σx)2/n)

The values are then added together

tn = sum of all (n)

tx = sum of all Σx

txx = sum of all Σx2

The combined calculations are

Combined n = tn

Combined mean = tx / tn

Combine SD = sqrt((txx-tx2/tn) / (tn-1))

**Supplemental figures and tables legends**

**Supplementary Fig.1 The effect size of the parameters of the elevated plus maze test (EPM) evaluating anxiety-like behaviours.**

**
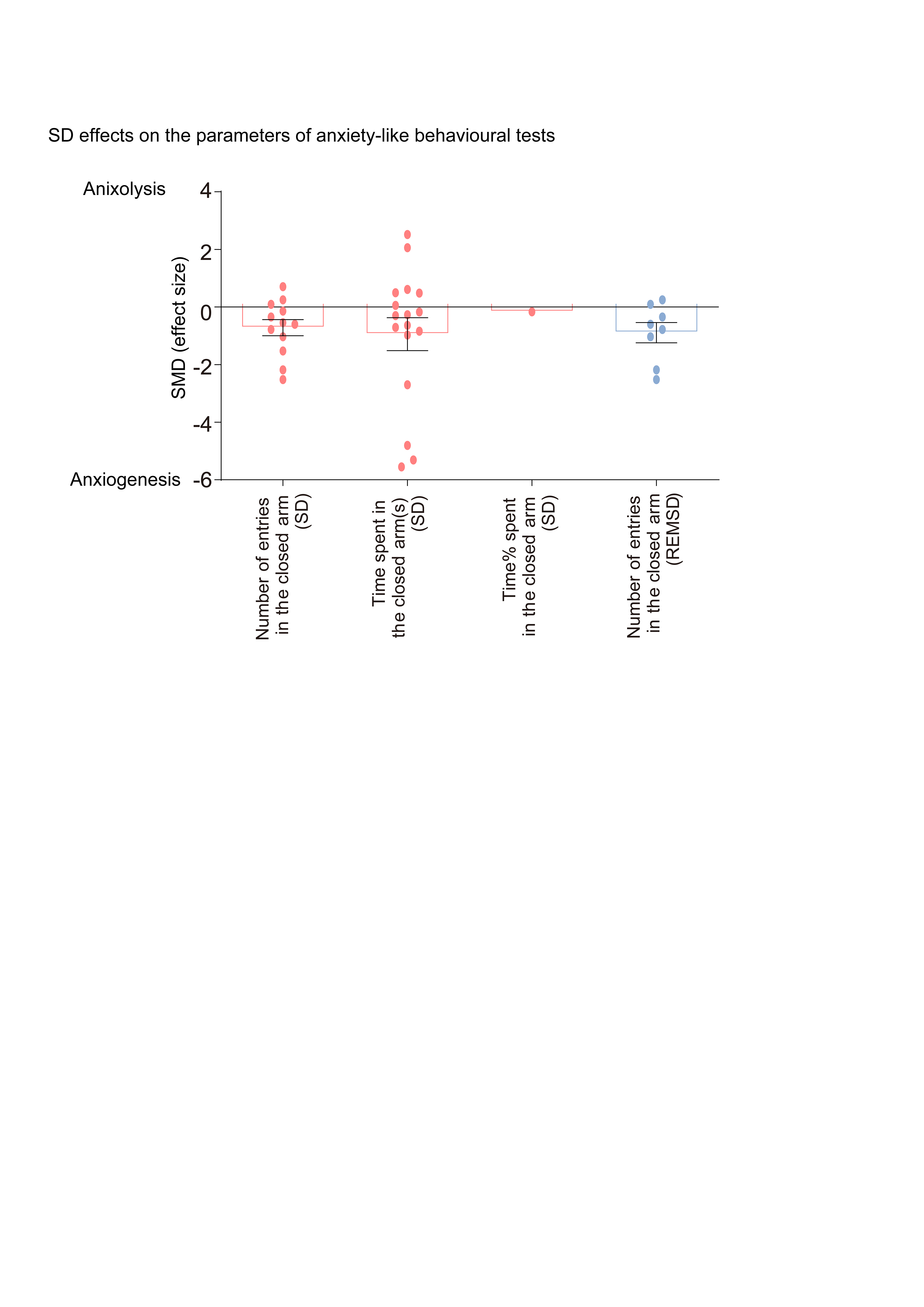
**

The effect size of the parameters was extracted from the meta-analysis of EPM test in cluster four (Supplementary Table 3). The difference was tested with one-way ANOVA, there were no significant difference on the parameters' sensitivity on detecting the effects of SD or REMSD among each parameter (F _(2, 27)_ = 0.111, *P* = 0.896). Number of entries in the closed arm (SD), n =12; Time spent in the closed arm(s) (SD), n =17; Time% spent in the closed arm (SD), n =1; Number of entries

in the closed arm (REMSD), n =8. Each dot represents the effect size generated from one experiment. n: sample size; REMSD: rapid eye movement sleep deprivation; SD: sleep deprivation.

**Supplementary Fig.2 The effect size of the parameters of the elevated plus maze test (EPM) between rodent species (A) and sleep deprivation duration (B).**

**
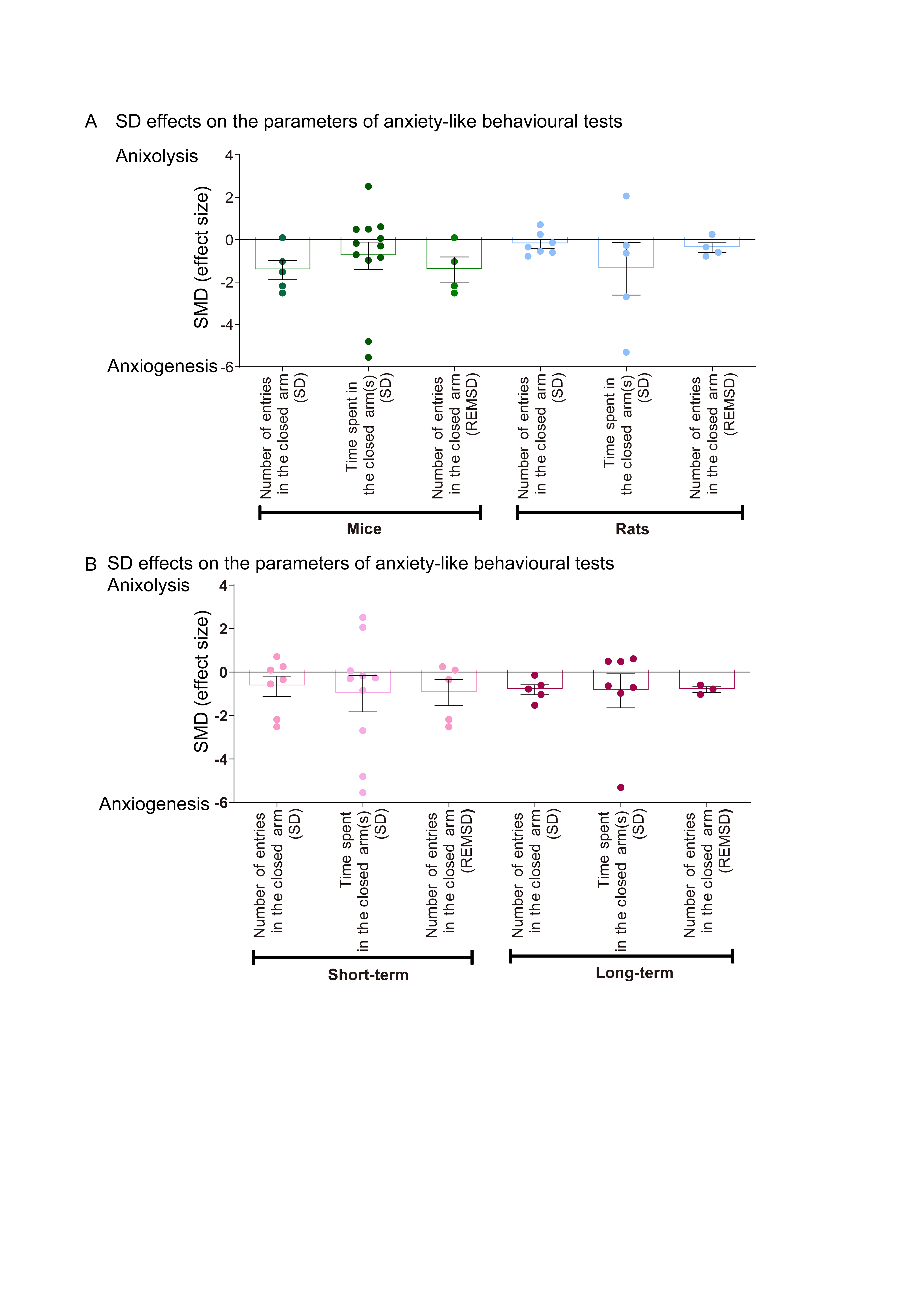
**

The difference was tested with two-way ANOVA, there were no significant difference on the parameters' sensitivity on species (A: parameters, F _(2, 31)_ = 0.513, *P* = 0.726; species, F _(1, 31)_ = 0.685, *P* = 0.414) and sleep deprivation duration (B: parameters, F _(2, 31)_ = 0.040, *P* = 0.961; duration, F _(1, 31)_ = 0.003, *P* = 0.957) among each parameter. The effect size of the parameters was extracted from the meta-analysis of EPM test in cluster four (Supplementary Table 3). A: Mice: Number of entries in the closed arm (SD), n =5; Time spent in the closed arm(s) (SD), n =12; Number of entries in the closed arm (REMSD), n =4; Rats: Number of entries in the closed arm (SD), n =7; Time spent in the closed arm(s) (SD), n =5; Number of entries in the closed arm (REMSD), n =4. B: Short-term: Number of entries in the closed arm (SD), n =7; Time spent in the closed arm(s) (SD), n =10; Number of entries in the closed arm (REMSD), n =5; Long-term: Number of entries in the closed arm (SD), n =5; Time spent in the closed arm(s) (SD), n =7; Number of entries in the closed arm (REMSD), n =3. Each dot represents the effect size generated from one experiment. n: sample size; REMSD: rapid eye movement sleep deprivation; SD: sleep deprivation.

**Supplementary Table 1 Search strategies in the Scopus and Embase database.**

| **Database** | **Search terms** |
| --- | --- |
| **Scopus** | TITLE ( "sleep deprivation" OR "sleep restriction" OR "Deprivation, Sleep " OR "REM Sleep Deprivation " OR "Deprivation, REM Sleep" OR "Sleep Deprivation" OR "REM，Sleep Insufficiency" OR "Insufficiencies, Sleep" OR "Insufficiency, Sleep" OR "Sleep Insufficiencies" OR "Insufficient Sleep" OR "Sleep, Insufficient" OR "Inadequate Sleep" OR "Sleep, Inadequate" OR "Sleep Fragmentation" OR "Fragmentation, Sleep" OR "Insufficient Sleep Syndrome" OR "Insufficient Sleep Syndromes" OR "Syndrome, Insufficient Sleep" OR "Sleep Debt" ) AND ALL ( "Rodent" OR "Rat" OR "Mus" OR "Mouse" OR "Mus domesticus" OR "Mus musculus domesticus" OR "domesticus, Mus musculus" OR "Mus musculus" OR "Mice, House" OR "House Mice" OR "Mouse, House" OR "House Mouse" OR "Mouse, Swiss" OR "Swiss Mouse" OR "Swiss Mice" OR "Mice, Swiss" OR "Mice, Laboratory" OR "Laboratory Mice" OR "Mouse, Laboratory" OR "Laboratory Mouse" ) AND LANGUAGE ( english ) |
| **Embase** | (('sleep restriction':ti OR deprivation:ti) AND sleep:ti OR 'rem sleep deprivation':ti OR 'deprivation , rem sleep':ti OR 'sleep deprivation':ti OR 'rem, sleep insufficiency':ti OR 'insufficiencies, sleep':ti OR 'insufficiency, sleep':ti OR 'sleep insufficiencies':ti OR 'insufficient sleep':ti OR 'sleep, insufficient':ti OR 'inadequate sleep':ti OR 'sleep, inadequate':ti OR 'sleep fragmentation':ti OR 'fragmentation, sleep':ti OR 'insufficient sleep syndrome':ti OR 'insufficient sleep syndromes':ti OR 'syndrome, insufficient sleep':ti OR 'sleep debt':ti) AND ('rodent' OR 'rat' OR 'mus' OR 'mouse' OR 'mus domesticus' OR 'mus musculus domesticus' OR 'domesticus, mus musculus' OR 'mus musculus' OR 'mice, house' OR 'house mice' OR 'mouse, house' OR 'house mouse' OR 'mouse, swiss' OR 'swiss mouse' OR 'swiss mice' OR 'mice, swiss' OR 'mice, laboratory' OR 'laboratory mice' OR 'mouse, laboratory' OR 'laboratory mouse') AND [english]/lim AND [animals]/lim |

**Supplementary Table 2 List and description of selected articles.**

| **Article** | **Species/** **congenic background** | **Gender** | **SD manipulation** | **SD duration** | **Time interval between behavioural tests** | **Behavioural test** |
| --- | --- | --- | --- | --- | --- | --- |
| Abayomi et al., 2022 | Wistar Rats | Male | REMSD (MMP) | 21h/day for 1 weeks (Long-erm) | No information | EPM, OF |
| Abbasy et al., 2021 | Wistar Rats | Male | Non-REMSD (Moved Pedal) | 48h (Short-term) | No information | FC, FST, OF |
| Abd Rashid et al., 2017 | Sprague Dawley Rats | Male | REMSD (MMP) | 72h (Short-term) | No information | MWM |
| Ahmed et al., 2021 | C57BL/6J Mice | Male &Female | Non-REMSD (Treadmill) | 24h (Short-term) | No information | EPM, FST, OF, SPT, TST |
| Aleisa et al., 2011 | Wistar Rats | Male | REMSD (MMP) | 24h,48h (Short-term) | No information | RAWM |
| Alhaider et al., 2009 | Wistar Rats | Male | REMSD (MMP) | 24h (Short-term) | No information | RAWM |
| Alhaider et al., 2011 | Wistar Rats | Male | REMSD (MMP) | 24h (Short-term) | No information | RAWM |
| Alvarenga et al., 2008 | Wistar Rats | Male | REMSD (MMP) | 96h (Long-term) | No information | PMDAT |
| Alzoubi et al., 2012 | Wistar Rats | Male | REMSD (MMP) | 8h/day for 6 weeks (Long-term) | No information | RAWM |
| Alzoubi er al., 2015 | Wistar Rats | Male | REMSD (MMP) | 8h/day for 4 weeks (Long-term) | No information | RAWM |
| Alzoubi et al., 2016 | Wistar Rats | Male | REMSD (MMP) | 8h/day for 6 weeks (Long-term) | No information | OF, RAWM |
| Alzoubi et al., 2017a | Wistar Rats | Male | REMSD (MMP) | 8h/day for 6 weeks (Long-term) | During the SD procedure | RAWM |
| Alzoubi et al., 2017b | Wistar Rats | Male | REMSD (MMP) | 8h/day for 8 weeks (Long-term) | No information | RAWM |
| Alzoubi et al., 2019a | Wistar Rats | Male | REMSD (MMP) | 8h/day for 4 weeks (Long-term) | No information | RAWM |
| Alzoubi et al., 2019b | Wistar Rats | Male | REMSD (MMP) | 8h/day for 8 weeks (Long-term) | No information | RAWM |
| Alzoubi et al., 2020 | Wistar Rats | Male | REMSD (MMP) | 8h/day for 4 weeks (Long-term) | During the SD procedure | RAWM |
| Araujo et al., 2006 | Swiss EPM-M1Mice | Male &Female | REMSD (MMP) | 48h (Short-term) | During the SD procedure | OF |
| Arora et al., 2021 | Swiss albino Mice | Female | REMSD (MMP) | 8h/day for 5days, 9 weeks (Long-term) | During the SD procedure | FST, NOR, OF, SPT, EPM |
| Arvin et al., 2022 | Wistar Rats | Male | Non-REMSD (Electric shock) | 18h/day, 21days (Long-term) | No information | FST, PAT |
| Baratta et al., 2018 | Wistar Rats | Male &Female | Non-REMSD (Gentle handle) | 6h (Short-term) | No information | NOR |
| Barbosa et al., 2019 | Nulliparous Swiss Mice | Female | Non-REMSD (Sleep restriction) | 2h/day for 15 days (Long-term) | No information | PAT |
|  |  |  | REMSD (MMP) | 72h (Short-term) | No information | PAT |
| Brice et al., 2020 | C57BL/6J Mice | Male | Non-REMSD (Sleep restriction) | 10h/day for 6 weeks (Long-term) | No information | FC |
| Cakir et al., 2020 | Wistar Rats | Male | REMSD (MMP) | 96h (Long-term) | No information | MWM |
| Chang et al., 2009 | Wistar Rats | Male | Non-REMSD (DOW) | 72h (Short-term) | No information | MWM |
| Chen et al., 2014 | Sprague Dawley Rats | Male | REMSD (MMP) | 6h (Short-term) | During the SD procedure | NOR |
| Chen et al., 2020 | Sprague Dawley Rats | Male | REMSD (MMP) | 72h (Short-term) | Less than two hours | MWM |
| Cordeira et al., 2018 | C57BL/6 Mice | Female | Non-REMSD (Rotating wheels) | 12h (Short-term) | Less than two hours | NOR |
| Daniele et al., 2017 | Swiss Mice | Male | Non-REMSD (Gentle handle) | 6h (Short-term) | Less than two hours | EPM, TST |
| Davis et al., 2021 | Long Evans Rats | Male | Non-REMSD (Gentle handle) | 12h (Short-term) | No information | FC |
| de Oliveira et al., 2004 | Wistar Rats | Female | REMSD (MMP) | 72h (Short-term) | Less than two hours | EPM, OF, PAT, FST, |
| Delorme et al., 2021 | C57BL/6J Mice | No information | Non-REMSD (Gentle handle) | 6h (Short-term) | No information | FC |
| Dhaliwal et al., 2018 | Swiss albino Mice | Male | Non-REMSD (Metal grid) | 72h (Short-term) | No information | MWM, FST, TST, PAT |
| Di Meco et al., 2014 | C57BL/6 Mice | No information | Non-REMSD (Sleep restriction) | 20h/day for 8 weeks (Long-term) | No information | FC, MWM |
| Dos Santos et al., 2013 | Wistar Rats | Male | REMSD (MMP) | 24h (Short-term) | No information | NOR, OF, |
| Duan et al., 2016 | Wistar Rats | Male | REMSD (MMP) | 72h (Short-term) | No information | MWM |
| Dubiela et al., 2010 | Wistar Rats | Male | REMSD (MMP) | 96h (Long-term) | No information | PAT |
| Dubiela et al., 2011 | Wistar Rats | Male | REMSD (MMP) | 96h (Long-term) | No information | EPM, OF |
| Dubiela et al., 2013 | Wistar Rats | Male | REMSD (MMP) | 96h (Long-term) | No information | PAT |
| Esmaeilpour et al., 2015 | Wistar Rats | Female | REMSD (MMP) | 72h (Short-term) | No information | MWM |
| Fernandes-Santos et al., 2012 | Swiss Mice | Male &Female | Non-REMSD (Gentle handle) | 6h (Short-term) | Less than two hours | PMDAT, PAT, FC |
| Frolinger et al., 2018 | C57BL/6J Mice | Male | Non-REMSD (Rotating wheels) | 5h (Short-term) | Twenty-four hours | NLR, NOR |
| Gao et al., 2020 | Sprague Dawley Rats | Male | Non-REMSD (Intermittent tactile stimulation) | 48h (Short-term) | No information | OF |
| Gao et al., 2021 | Wistar Rats | Male | REMSD (MMP) | 72h (Short-term) | No information | NOR, MWM |
| Gonzalez-Castañeda et al., 2016 | BALB/c Mice | Male | REMSD (MMP) | 48h (Short-term) | Less than two hours | EPM, FST, OF, SPT |
| Graves et al., 2003 | C57BL/6J Mice | Male & Female | Non-REMSD (Gentle stroking) | 5h (Short-term) | No information | FC |
| Grubac et al., 2019 | Wistar Rats | Male | Non-REMSD (Treadmill) | 18h (Short-term) | Less than two hours | EPM, OF |
| Gu et al. 2022 | Wistar Rats | Male | REMSD (MMP) | 7 days (Long-term) | Less than two hours | SPT, FST, TST |
| Guan et al., 2004 | Sprague Dawley Rats | Male | Non-REMSD (Novel objects stimulate) | 6h (Short-term) | During the SD procedure | MWM |
| Guo et al., 2021 | Rats (No information on strains) | Male | REMSD (MMP) | 48h (Short-term) | No information | MWM |
| Hagewoud et al., 2010 | Wistar Rats | Male | Non-REMSD (Mild stimulation) | 12h (Short-term) | During the SD procedure | FC |
| Hagewoud et al., 2011 | Wistar Rats | Male | Non-REMSD (Mild stimulation) | 6h (Short-term) | During the SD procedure | FC |
| Hajali et al., 2012 | Wistar Rats | Male | REMSD (MMP) | 72h (Short-term) | Less than two hours | MWM, OF |
| Hajali et al., 2015 | Sprague Dawley Rats | Male | REMSD (MMP) | 48h (Short-term) | No information | MWM |
| Hakimeh et al., 2017 | Wistar Rats | Female | REMSD (MMP) | 72h (Short-term) | No information | OF |
| Han et al., 2017 | Wistar Rats | Male | REMSD (MMP) | 14h/day for 5 days, (Long-term) | Less than two hours | EPM, OF |
|  |  |  | REMSD (MMP) | 14h/day for 14 days, (Long-term) | Less than two hours | EPM, OF |
|  |  |  | REMSD (MMP) | 14h/day for 21days, (Long-term) | Less than two hours | EPM, OF |
| Han et al., 2018 | Wistar Rats | Male | REMSD (MMP) | 14h/day for 21days (Long-term) | No information | EPM, OF |
| He et al., 2015 | C57BL Mice | Male | Non-REMSD (Sleep Fragmentation) | 10days (Long-term) | During the SD procedure | EPM |
| Hicks et al., 1979 | Sprague Dawley Rats | Male | REMSD (MMP) | 48h (Short-term) | No information | OF |
|  |  |  | REMSD (MMP) | 96h (Long-term) | No information | OF |
| Hines et al., 2013 | Mice (No information on strains) | Male | Non-REMSD (Randomly timed movements) | 12h (Short-term) | No information | FST, TST |
| Hou et al., 2019 | Sprague Dawley Rats | Male | REMSD (MMP) | 96h (Long-term) | Twenty-four hours | MWM |
| Hunter et al., 2018 | Rats (No information on strains) | Male | Non-REMSD (Sleep fragmentation) | 6h (Short-term) | Twenty-four hours | FC |
|  |  |  | REMSD (MMP) | 6h (Short-term) | Twenty-four hours | FC |
| Ishikawa et al, 2014 | Wistar Rats | Male | REMSD (MMP) | 4h (Short-term) | No information | NLR, NOR |
| Jiang et al., 2015 | Sprague Dawley Rats | No information | REMSD (MMP) | 48h (Short-term) | No information | SPT |
| Jiao et al., 2022 | C57BL/6J Mice | Male | REMSD (MMP) | 24h (Short-term) | No information | NLR, NOR |
|  |  |  | REMSD (MMP) | 48h (Short-term) | No information | NLR, NOR |
|  |  |  | REMSD (MMP) | 72h (Short-term) | No information | NLR, NOR |
| Kalonia et al., 2008 | Wistar Rats | Male | REMSD (MMP) | 72h (Short-term) | No information | PAT |
| Kang et al., 2021 | Wistar Rats | Male | REMSD (MMP) | 72h (Short-term) | Less than two hours | EPM, FST, OF, TST |
| Karabulut et al., 2019 | BALB/c Mice | Male | REMSD (MMP) | 6h (Short-term) | During the SD procedure | MWM |
| Kaur et al., 2017 | Wistar Rats | Female | Non-REMSD (Gentle handle) | 12h (Short-term) | Less than two hours | TST, OF, FST, EPM |
| Kinchski et al., 2017 | Swiss Mice | Male | Non-REMSD (Gentle handle) | 3h/day,5days/weeks, 30weeks (Long-term) | Twenty-four hours | FC |
|  |  |  | REMSD (MMP) | 72h (Short-term) | Twenty-four hours | FC |
| Kordestani-Moghadam et al., 2020 | Wistar Rats | Male | Non-REMSD (Rotation) | 48h (Short-term) | No information | FST |
|  |  |  | REMSD (MMP) | 48h (Short-term) | No information | FST |
| Kumar et al., 2009 | Swiss albino mice | Male &Female | REMSD (MMP) | 72h (Short-term) | No information | EPM |
| Kumar et al., 2012 | Wistar Rats | Male | Non-REMSD (Gentle handle) | 6h (Short-term) | During the SD procedure | FC |
| Lu et al., 2021 | ICR Mice | Male | Non-REMSD (Rotation) | 18h/day for 21 days (Long-term) | Less than two hours | MWM |
| Lu et al., 2023 | Sprague Dawley Rats | Male | REMSD (MMP) | 96h (Long-term) | Less than two hours | MWM |
| Mahboubi et al., 2019 | Wistar Rats | Male | REMSD (MMP) | 24h (Short-term) | No information | MWM |
| Maher et al., 2021 | Swiss albino mice | Male | REMSD (MMP) | 12h/day for 6 days (Long-term) | No information | MWM |
| Manchanda et al., 2017 | Wistar Rats | Female | Non-REMSD (Gentle handle) | 12h (Short-term) | Less than two hours | NOR |
| Manchanda et al., 2018 | Wistar Rats | Male | Non-REMSD (Rotation) | 12h/day for 21days (Long-term) | Less than two hours | EPM, OF |
| Martinez-Gonzalez et al., 2004 | Long Evans Rats | Male | REMSD (MMP) | 5 days (Long-term) | No information | EPM, OF |
| Massadeh et al., 2022a | Wistar Rats | Male | REMSD (MMP) | 8h/day for 6 weeks (Long-term) | No information | RAWM |
| Massadeh et al., 2022b | Wistar Rats | Male | REMSD (MMP) | 8h/day for 6 weeks (Long-term) | During the SD procedure | RAWM |
| Matos et al., 2012 | Wistar Rats | Male | REMSD (MMP) | 24h (Short-term) | Less than two hours | OF |
| Maturana et al., 2015 | Wistar Rats | Male | REMSD (MMP) | 72h (Short-term) | Less than two hours | FST, OF |
| Mhaidat et al., 2015 | Wistar Rats | Male | REMSD (MMP) | 8h/day for 6 weeks (Long-term) | Less than two hours | RAWM |
| Mishra et al., 2016 | Wistar Rats | Female | Non-REMSD (Gentle handle) | 12h (Short-term) | Less than two hours | EPM, NOR, OF |
| Misrani et al., 2019 | C57BL/6J Mice | Male & Female | Non-REMSD (Treadmill) | 24h (Short-term) | No information | EPM, FST, MWM, OF, SPT, TST |
| Mohammadipoor-Ghasemabad et al., 2019 | Wistar Rats | Female | REMSD (MMP) | 72h (Short-term) | Less than two hours | MWM |
| Moreira et al., 2003 | Wistar Rats | Male | REMSD (MMP) | 96h (Long-term) | Less than two hours | PAT |
| Murack et al., 2021 | CD-1 Mice | Male & Female | Non-REMSD (Gentle handle) | 4h/day for 8 days (Long-term) | No information | FST |
| Nabaee et al., 2018 | Wistar Rats | Male | REMSD (MMP) | 24h (Short-term) | No information | PAT |
|  |  |  | REMSD (MMP) | 48h (Short-term) | No information | PAT |
|  |  |  | REMSD (MMP) | 72h (Short-term) | No information | PAT |
| Nair et al., 2011 | C57BL/6J Mice | Male | Non-REMSD (Sleep Fragmentation) | 14 days (Long-term) | Less than two hours | EPM, MWM |
| Nasehi et al., 2018 | Wistar Rats | Male | REMSD (MMP) | 24h (Short-term) | Less than two hours | FC, FST |
| Noorafshan et al., 2017 | Sprague Dawley Rats | Male | REMSD (MMP) | 18h/day for 21days (Long-term) | No information | NOR |
| Novozhilova et al., 2021 | C57BL/6 Mice | Female | REMSD (MMP) | 10h/day for 5 days (Long-term) | Less than two hours | MWM, OF |
| Ocalan et al., 2019 | Wistar Rats | Male | REMSD (MMP) | 96h (Long-term) | No information | MWM |
| Orozco-Solis et al., 2017 | C57BL/6J Mice | Male | Non-REMSD (Rotation) | 12h (Short-term) | Less than two hours | FST |
| Oyanedel et al., 2014 | Long Evans Rats | Male | Non-REMSD (Gentle handle) | 5h (Short-term) | No information | NOR， NLR |
| Özakman et al., 2021 | BALB/c Mice | Male | REMSD (MMP) | 24h (Short-term) | No information | NOR |
| Patti et al., 2010 | Swiss Mice | Male | Non-REMSD (Gentle handle) | 72h (Short-term) | Less than two hours | PMDAT |
| Pittaras et al., 2022 | DBA/2J Mice | Male | Non-REMSD (Gentle handle) | 4h (Short-term) | Less than two hours | NLR, NOR |
| Proença et al., 2014 | Wistar Rats | Male | REMSD (MMP) | 24h (Short-term) | Twenty-four hours | OF, NOR |
| Puech et al., 2023 | C57BL/6J Mice | Male | Non-REMSD (Sleep Fragmentation) | 12h/day for 4 weeks (Long-term) | No information | NOR |
| Qiu et al., 2021 | C57BL/6 Mice | Male | REMSD (MMP) | 20h/day for 28 days (Long-term) | No information | MWM, NOR |
| Rahimpour et al., 2023 | Wistar Rats | Male | Non-REMSD (Rotation) | 24h (Short-term) | No information | MWM |
| Rajizadeh et al., 2018 | Wistar Rats | Female | REMSD (MMP) | 72h (Short-term) | Less than two hours | NOR, MWM, PAT, OF |
| Rajizadeh et al., 2019 | Wistar Rats | Male | REMSD (MMP) | 72h (Short-term) | Twenty-four hours | MWM, OF |
| Rajizadeh et al., 2020 | Wistar Rats | Female | REMSD (MMP) | 72h (Short-term) | Less than two hours | NLR |
| Rezaie et al., 2021 | Wistar Rats | Male | Non-REMSD (Rotation) | 24h (Short-term) | No information | FST |
| Rosier et al., 2018 | C57BL/6J Mice | Male | REMSD (Shanking under EEG and EMG monitor) | 6h (Short-term) | During the SD procedure | FC |
| Ruskin et al., 2004 | Sprague Dawley Rats | Male | REMSD (MMP) | 72h (Short-term) | Less than two hours | FC |
| Ruskin et al., 2008 | Sprague Dawley Rats | Male | REMSD (MMP) | 72h (Short-term) | During the SD procedure | FC |
|  |  |  | Non-REMSD (sleep restriction) | 20h/day for 3 days (Short-term) | During the SD procedure | FC |
| Saadati et al., 2014 | Wistar Rats | Female | REMSD (MMP) | 72h (Short-term) | Less than two hours | MWM |
| Salari et al., 2015 | Wistar Rats | Female | REMSD (MMP) | 24h (Short-term) | Less than two hours | MWM |
| Salberg et al., 2018 | Sprague Dawley Rats | Male &Female | Non-REMSD (Gentle handle) | 5h (Short-term) | Twenty-four hours | EPM, OF, FST |
| Santos et al., 2016 | Wistar Rats | Male | REMSD (MMP) | 24h (Short-term) | Less than two hours | NOR |
|  |  |  | REMSD (MMP) | 48h (Short-term) | Less than two hours | NOR |
|  |  |  | REMSD (MMP) | 72h (Short-term) | Less than two hours | NOR |
|  |  |  | REMSD (MMP) | 96h (Long-term) | Less than two hours | NOR |
| Saré et al., 2016 | C57BL/6J Mice | Male | Non-REMSD (Sleep restriction) | 3h/day for 30 days (Long-term) | Twenty-four hours | EPM, OF |
| Shahveisi et al., 2020 | Wistar Rats | Male | REMSD (MMP) | 12h (Short-term) | Less than two hours | NOR |
| Sharma et al., 2020 | C57BL/6J Mice | Male | Non-REMSD (Gentle handle) | 5h (Short-term) | During the SD procedure | FC |
| Shen et al., 2021 | Sprague Dawley Rats | Male | REMSD (MMP) | 24h (Short-term) | Less than two hours | MWM |
| Siddique et al., 2018 | Swiss albino Mice | Male | REMSD (MMP) | 48h (Short-term) | Less than two hours | FST, OF |
|  |  |  | REMSD (MMP) | 72h (Short-term) | Less than two hours | FST, OF |
|  |  |  | REMSD (MMP) | 96h (Long-term) | Less than two hours | FST, OF |
| Silva et al., 2004a | Swiss EPM-M1Mice | Male | REMSD (MMP) | 72h (Short-term) | Less than two hours | PAT |
| Silva et al., 2004b | Swiss EPM-M1Mice | Male | REMSD (MMP) | 72h (Short-term) | No information | EPM, OF |
| Silva et al., 2004c | Swiss EPM-M1Mice | Male | REMSD (MMP) | 72h (Short-term) | No information | PAT |
| Silva et al., 2007 | Swiss EPM-M1Mice | Male | REMSD (MMP) | 24h (Short-term) | No information | PAT |
| Singh et al., 2007 | Swiss albino mice | Male | REMSD (MMP) | 72h (Short-term) | No information | EPM |
| Solanki et al., 2016 | Long Evans Rats | Male | REMSD (MMP) | 24h (Short-term) | Less than two hours | EPM, FST, OF, RAWM |
| Su et al., 2016 | Wistar Rats | Male | REMSD (MMP) | 72h (Short-term) | Less than two hours | OF, MWM |
| Suchecki et al., 2002 | Wistar Rats | Male | REMSD (MMP) | 96h (Long-term) | Less than two hours | EPM |
| Tabassum et al., 2019 | C57BL/6J Mice | Female | Non-REMSD (Treadmill) | 72h (Short-term) | During the SD procedure | MWM |
| Tai et al., 2020 | C57BL/6J Mice | Male | REMSD (MMP) | 8h/day for 8 weeks (Long-term) | No information | EPM, OF, MWM |
| Takatsu-Coleman et al., 2013 | Swiss Mice | Male | REMSD (MMP) | 12h (Short-term) | No information | PMDAT |
|  |  |  | REMSD (MMP) | 24h (Short-term) | No information | PMDAT |
| Tang et al., 2020 | Wistar Rats | Male | Non-REMSD (Rotation) | 3h/day for 14 days (Long-term) | No information | NLR, NOR, OF |
| Thomasson et al., 2017 | C57BL/6J Mice | Male | Non-REMSD (Sleep restriction) | 20h (Short-term) | No information | EPM |
| Tian et al., 2009 | Sprague Dawley Rats | Male | REMSD (MMP) | 6h (Short-term) | Twenty-four hours | FC |
| Tiba et al., 2008 | Wistar Rats | Male | REMSD (MMP) | 96h (Long-term) | Less than two hours | FC |
| Torabi et al., 2022 | Wistar Rats | Male | Non-REMSD (Rotation) | 24h (Short-term) | Twenty-four hours | EPM |
| Tseng et al., 2020 | C57BL/6J Mice | Male | Non-REMSD (Rotation) | 8h (Short-term) | No information | OF, EPM |
| Turan et al., 2021 | Wistar Rats | Male | REMSD (MMP) | 24h (Short-term) | During the SD procedure | EPM, FST, MWM, OF |
|  |  |  | REMSD (MMP) | 48h (Short-term) | During the SD procedure | EPM, FST, MWM, OF |
|  |  |  | REMSD (MMP) | 72h (Short-term) | During the SD procedure | EPM, FST, MWM, OF |
| Ugalde-Muñiz et al., 2022 | ICR Mice | Male | REMSD (MMP) | 72h (Short-term) | During the SD procedure | EPM, NOR, SPT |
| Vacas et al., 2017 | C57BL/6J Mice | Male | Non-REMSD (Sleep Fragmentation) | 24h (Short-term) | Twenty-four hours | FC |
| Vollert et al., 2011 | Wistar Rats | Male | REMSD (MMP) | 24h (Short-term) | Less than two hours | OF |
| Wallace et al., 2015 | C57BL/6 Mice | Male | Non-REMSD (sleep fragmentation) | 12h/day, 2 weeks (Long-term) | No information | MWM |
|  |  |  |  | 72h (Short-term) | No information | MWM |
| Walsh et al., 2011 | Sprague Dawley Rats | Male | REMSD (MMP) | 6h (Short-term) | No information | MWM |
| Wan et al., 2022 | C57BL/6 Mice | Male | REMSD (MMP) | 20h/day for 5 days (Long-term) | No information | MWM |
| Wang C et al., 2021 | C57BL/6J Mice | Male | REMSD (MMP) | 20/day for 21 days (Long-term) | No information | FC, MWM |
| Wang Z et al., 2017 | C57BL/6J Mice | Male | REMSD (MMP) | 5 days (Long-term) | No information | OF, EPM, FST, SPT |
|  |  |  | REMSD (MMP) | 72h (Short-term) | No information | OF, EPM, FST, SPT |
| Ward et al., 2017 | Sprague Dawley Rats | Male | Non-REMSD (Rotation) | 24h (Short-term) | No information | MWM |
| Xie et al., 2020 | C57BL/6J Mice | Male | Non-REMSD (sleep fragmentation) | 12h/day for 2 months (Long-term) | During the SD procedure | FST, MWM, NOR, OF |
| Xin et al., 2021 | C57BL/6 Mice | Male | Non-REMSD (Gentle handle) | 8h (Short-term) | Less than two hours | OF, FC |
| Xu Z et al., 2010 | C57BL/6 Mice | Female | Non-REMSD (Gentle handle) | 20 days (Short-term) | No information | MWM |
| Xu et al., 2021 | Sprague Dawley Rats | Male | REMSD (MMP) | 72h (Short-term) | Less than two hours | MWM |
| Xue et al., 2019 | C57BL/6 Mice | Male | REMSD (MMP) | 20h/day for 7 days (Long-term) | No information | MWM |
| Yan et al., 2022 | Sprague Dawley Rats | Male | REMSD (MMP) | 16h/day,4 weeks (Long-term) | No information | MWM |
| Yang et al., 2008 | Sprague Dawley Rats | Male | REMSD (MMP) | 72h (Short-term) | No information | MWM |
| Yang et al., 2019 | C57BL/6J Mice | Male | REMSD (MMP) | 72h (Short-term) | No information | MWM, NOR, NLR |
| Zagaar et al., 2012 | Wistar Rats | Male | REMSD (MMP) | 24h (Short-term) | No information | RAWM |
| Zager et al., 2009 | C57BL/6J Mice | Male | REMSD (MMP) | 72h (Short-term) | Less than two hours | EPM, OF |
| Zhang et al., 2013 | Sprague Dawley Rats | Male | REMSD (MMP) | 24h (Short-term) | No information | MWM, OF |
| Zhang et al., 2017 | C57BL/6J Mice | Male | REMSD (MMP) | 72h (Short-term) | No information | EPM, NLR, NOR, OF |
| Zhao et al., 2014 | Sprague Dawley Rats | Male | REMSD (MMP) | 72h (Short-term) | No information | MWM |
| Zhao et al., 2015 | C57BL/6J Mice | No information | Non-REMSD (Gentle handle) | 5h (Short-term) | No information | FC |
| Zhao et al., 2017 | C57BL/6 Mice | Male | Non-REMSD (sleep restriction) | 20h/day for 7 days (Long-term) | No information | MWM |
| Zhao et al, 2019 | Sprague Dawley Rats | Male | Non-REMSD (Rotation) | 20h/day for 14 days (Long-term) | No information | MWM |
| Zhu et al., 2023 | C57BL/6J Mice | Male | REMSD (MMP) | 20h/day for 14 days (Long-term) | No information | OF, NOR |

SD: sleep deprivation; REMSD: rapid eye movement sleep deprivation; Non-REMSD: a mix group of total sleep deprivation (TSD), sleep restriction (SR) and sleep fragmentation(SF); MMP: columns-in-water (modified multiple platforms); DOW: disc-on water method; FC: fear conditioning test; MWM: morris water maze test; NOR: novel object recognition test; NLR: novel location recognition test; PAT: passive avoidance test; PMDAT: plus-maze discriminative avoidance task; RAWM: radial arm water maze test; FST: forced swim test; SPT: sucrose preference test; TST: tail suspend test; EPM: elevated plus maze test; OF: open field test.

**Supplementary Table 3 Description and results on the meta-analysis of individual behavioural test in cluster four (including the data presented in Fig.7 and Supplementary Fig. 1 & 2)**

|  |  | **Exp** | **Con-*N*** | **Exp-*N*** | **Effect Estimate: SMD (95%)** | ***P*** |
| --- | --- | --- | --- | --- | --- | --- |
| **Overall analysis on memory function** | | | | | | |
| ***Fear conditioning test (FC)*** | | | | | | |
| #1 | SD effects | 34 | 330 | 338 | -1.07 [-1.41, -0.73] | 0.000^∗∗∗^ |
| #2 | REMSD effects | 12 | 100 | 100 | -1.06 [-1.62, -0.49] | 0.000^∗∗∗^ |
| Effects of SD on different parameters | | | | | | |
| #3 | Freezing% (context fear) | 25 | 243 | 250 | -1.06 [-1.47, -0.65] | 0.000^∗∗∗^ |
| #4 | Freezing% (cue fear) | 9 | 87 | 88 | -1.10 [-1.70, -0.50] | 0.003^∗∗^ |
| ***Morris water maze test (MWM)*** | | | | | | |
| #5 | SD effects | 101 | 986 | 987 | -1.38 [-1.63, -1.14] | 0.000^∗∗∗^ |
| #6 | REMSD effects | 76 | 701 | 707 | -1.45 [-1.76, -1.15] | 0.000^∗∗∗^ |
| Effects of SD on different parameters | | | | | | |
| #7 | Distance% in target quadrant | 13 | 102 | 104 | -1.35 [-1.89, -0.80] | 0.000^∗∗∗^ |
| #8 | Escape latency (s) | 24 | 244 | 235 | -0.83 [-1.36, -0.29] | 0.003^∗∗^ |
| #9 | Number of platform crossing | 21 | 232 | 240 | -1.79 [-2.34, -1.22] | 0.000^∗∗∗^ |
| #10 | Time spent in target quadrant (s) | 14 | 122 | 121 | -1.54 [-2.26, -0.82] | 0.000^∗∗∗^ |
| #11 | Time% in target quadrant | 29 | 286 | 287 | -1.51 [-1.92, -1.11] | 0.000^∗∗∗^ |
| ***Novel object recognition test (NOR)*** | | | | | | |
| #12 | SD effects | 35 | 427 | 429 | -1.22 [-1.53, -0.91] | 0.000^∗∗∗^ |
| #13 | REMSD effects | 23 | 315 | 315 | -1.32 [-1.74, -0.92] | 0.000^∗∗∗^ |
| Effects of SD on different parameters | | | | | | |
| #14 | Discrimination index (%) | 23 | 300 | 299 | -1.27 [-1.67, -0.87] | 0.000^∗∗∗^ |
| #15 | Recognition index (%) | 12 | 127 | 130 | -1.14 [-1.62, -0.65] | 0.000^∗∗∗^ |
| ***Novel location recognition test (NLR)*** | | | | | | |
| #167 | SD effects | 11 | 102 | 103 | -1.32 [-2.30, -0.35] | 0.008^∗∗^ |
| #17 | REMSD effects | 7 | 71 | 70 | -1.15 [-2.45, 0.18] | 0.090 |
| Effects of SD on different parameters | | | | | | |
| #18 | Discrimination index (%) | 9 | 87 | 86 | -1.36 [-2.53, -0.20] | 0.022^∗^ |
| #19 | Recognition index (%) | 2 | 15 | 17 | -1.17 [-2.95, 0.61] | 0.197 |
| ***Passive avoidance test (PAT)*** | | | | | | |
| #20 | SD effects | 23 | 188 | 198 | -0.81 [-1.33, -0.29] | 0.002^∗∗^ |
| #21 | REMSD effects | 18 | 138 | 146 | -0.94 [-1.40, -0.47] | 0.000^∗∗∗^ |
| Effects of SD on different parameters | | | | | | |
| #22 | Latency to enter dark compartment in long-term memory | 17 | 143 | 152 | -1.06 [-1.63, -0.49] | 0.000^∗∗∗^ |
| #23 | Latency to enter dark compartment in short-term memory | 6 | 45 | 46 | -0.06 [-1.28, 1.17] | 0.930 |
| ***Plus-maze discriminative avoidance task (PMDAT)*** | | | | | | |
| #24 | SD effects | 7 | 89 | 86 | -0.89 [-1.96, 0.18] | 0.103 |
| #25 | REMSD effects | 3 | 38 | 38 | 0.13 [ -1.20, 1.47] | 0.842 |
| Effects of SD on different parameters | | | | | | |
| #26 | Time spent in the aversive enclosed arms (s) | 4 | 56 | 54 | 0.04 [-0.96, 1.03] | 0.943 |
| #27 | Time% in aversive enclosed arm | 3 | 33 | 32 | -2.26 [-4.15, -0.37] | 0.019^∗^ |
| ***Radial arm water maze task (RAWM)*** | | | | | | |
| #28 | SD effects | 35 | 392 | 392 | -1.54 [-1.75, -1.32] | 0.000^∗∗∗^ |
| #29 | REMSD effects | 35 | 392 | 392 | -1.54 [-1.75, -1.32] | 0.000^∗∗∗^ |
| Effects of SD on different parameters | | | | | | |
| #30 | Number of errors long-term memory 24h | 7 | 76 | 76 | -1.50 [-2.07, -0.94] | 0.000^∗∗∗^ |
| #31 | Number of errors long-term memory 5h | 11 | 130 | 130 | -1.33 [-1.61, -1.06] | 0.000^∗∗∗^ |
| #32 | Number of errors short-term memory 30min | 17 | 186 | 186 | -1.73 [-2.10, -1.36] | 0.000^∗∗∗^ |
| **Overall analysis on depression-like behaviours** | | | | | | |
| ***Forced swim test (FST)*** | | | | | | |
| #33 | SD effects | 35 | 345 | 353 | -1.12 [-1.64, -0.61] | 0.000^∗∗∗^ |
| #34 | REMSD effects | 20 | 207 | 207 | -1.56 [-2.27, -0.84] | 0.000^∗∗∗^ |
| Effects of SD on different parameters | | | | | | |
| #35 | Immobility (s) | 35 | 345 | 353 | -1.12 [-1.64, -0.61] | 0.000^∗∗∗^ |
| ***Sucrose preference test (SPT)*** | | | | | | |
| #32 | SD effects | 14 | 159 | 160 | -1.50 [-2.04, -0.97] | 0.000^∗∗∗^ |
| #33 | REMSD effects | 12 | 137 | 138 | -1.42 [-2.02, -0.83] | 0.000^∗∗∗^ |
| Effects of SD on different parameters | | | | | | |
| #34 | Sucrose preference% | 14 159 160 -1.50 [-2.04, -0.97] 0.000* | 159 160 -1.50 [-2.04, -0.97] 0.000* | 160 | -1.50 [-2.04, -0.97] | 0.000^∗∗∗^ |
| ***Tail suspends test (TST)*** | | | | | | |
| #35 | SD effects | 8 | 84 | 83 | -3.26 [-5.04, -1.48] | 0.000^∗∗∗^ |
| #36 | REMSD effects | 3 | 27 | 27 | -4.64 [-7.97, -1.30] | 0.006^∗∗^ |
| Effects of SD on different parameters | | | | | | |
| #37 | Immobility time (s) | 8 | 84 | 83 | -3.26 [-5.04, -1.48] | 0.000^∗∗∗^ |
| **Overall analysis on Anxiety-like behaviours** | | | | | | |
| ***Elevated plus maze test (EPM)*** | | | | | | |
| #38 | SD effects | 25 | 302 | 300 | -0.62 [-1.05, -0.20] | 0.004^∗∗^ |
| #39 | REMSD effects | 17 | 204 | 204 | -0.65 [-1.25, -0.05] | 0.034^∗^ |
| Effects of SD on different parameters | | | | | | |
| #40 | Number of entries in the closed arm | 12 | 106 | 106 | -0.64 [-1.11, -0.17] | 0.008^∗∗^ |
| #41 | Time spent in the closed arm (s) | 17 | 187 | 185 | -0.59 [-1.25, 0.06] | 0.007^∗∗^ |
| #42 | Time% spent in the closed arm | 1 | 9 | 9 | -1.67 [-2.76, -0.58] | 0.003^∗∗^ |
| Effects of REMSD on different parameters | | | | | | |
| #43 | Number of entries in the closed arm | 8 | 72 | 72 | -0.75 [-1.34, -0.16] | 0.013^∗^ |
| #44 | Time spent in the closed arm (s) | 12 | 132 | 132 | -0.62 [-1.54, 0.31] | 0.189 |
| ***Open field test (OF)*** | | | | | | |
| #45 | SD effects | 32 | 387 | 375 | -0.17 [-0.60, 0.27] | 0.451 |
| #46 | REMSD effects | 22 | 226 | 234 | 0.43 [-0.08, 0.94] | 0.097 |
| Effects of SD on different parameters | | | | | | |
| #47 | Immobility time (s) | 12 | 128 | 128 | -0.21 [-0.85, 0.43] | 0.516 |
| #48 | Time spent in center% | 6 | 88 | 67 | -0.67 [-1.57, 0.24] | 0.149 |
| #49 | Time spent in central area (s) | 16 | 171 | 180 | 0.10 [ -0.67, 0.86] | 0.802 |
| Effects of REMSD on different parameters | | | | | | |
| #50 | Immobility time (s) | 8 | 69 | 69 | 0.31 [-0.19, 0.81] | 0.233 |
| #51 | Time spent in center% | 2 | 27 | 26 | -0.09 [-2.43, 2.26] 2.260 | 0.943 |
| #52 | Time spent in central area (s) | 12 | 130 | 139 | 0.68 [-0.17, 1.53] | 0.115 |
| ***Plus-maze discriminative avoidance task (PMDAT)*** | | | | | | |
| #53 | SD effects | 4 | 46 | 44 | -0.48 [-1.08, 0.12] | 0.119 |
| #54 | REMSD effects | 1 | 8 | 8 | -1.58 [-2.72, -0.44]  ---------------------+------------------------------ | 0.007^∗∗^ |
| Effects of SD on different parameters | | | | | | |
| #55 | Time% spent in the open arm | 4 | 46 | 44 | -0.48 [-1.08, 0.12] | 0.119 |
| Effects of REMSD on different parameters | | | | | | |
| #56 | Time% spent in the open arm | 1 | 8 | 8 | -1.58 [-2.72, -0.44] | 0.007^∗∗^ |

SD: sleep deprivation; REMSD: rapid eye movement sleep deprivation; Exp: number of the experiments; Con-N: sample size of the control group; Exp-N: sample size of the SD intervention group. ∗ *P* ≤0.05, ∗∗ *P* ≤0.01, ∗∗∗ *P* ≤0.001

**Supplementary Table 4 Details of selected studies on total sleep deprivation, sleep restriction and sleep fragmentation**

| **Species** | **SD duration** | **Number of studies** |
| --- | --- | --- |
| **Memory function** | | |
| ***Fear conditioning test (FC)*** | | |
| Mice | Long-term | 3 |
|  | Short-term | 7 |
| Rats | Long-term | 0 |
|  | Short-term | 7 |
| ***Morris water maze test (MWM)*** | | |
| Mice | Long-term | 6 |
|  | Short-term | 6 |
| Rats | Long-term | 1 |
|  | Short-term | 4 |
| ***Novel object recognition test (NOR)*** | | |
| Mice | Long-term | 2 |
|  | Short-term | 3 |
| Rats | Long-term | 1 |
|  | Short-term | 5 |
| ***Novel location recognition test (NLR)*** | | |
| Mice | Long-term | 0 |
|  | Short-term | 2 |
| Rats | Long-term | 1 |
|  | Short-term | 1 |
| ***Passive avoidance test (PAT)*** | | |
| Mice | Long-term | 1 |
|  | Short-term | 2 |
| Rats | Long-term | 1 |
|  | Short-term | 0 |
| ***Plus-maze discriminative avoidance task (PMDAT)*** | | |
| Mice | Long-term | 0 |
|  | Short-term | 2 |
| Rats | Long-term | 0 |
|  | Short-term | 0 |
| **Depression-like behaviours** | | |
| ***Forced swim test (FST)*** | | |
| Mice | Long-term | 2 |
|  | Short-term | 6 |
| Rats | Long-term | 1 |
|  | Short-term | 5 |
| ***Sucrose preference test (SPT)*** | | |
| Mice | Long-term | 0 |
|  | Short-term | 2 |
| Rats | Long-term | 0 |
|  | Short-term | 0 |
| ***Tail suspends test (TST)*** | | |
| Mice | Long-term | 0 |
|  | Short-term | 5 |
| Rats | Long-term | 0 |
|  | Short-term | 1 |
| **Anxiety-like behaviours** | | |
| ***Elevated plus maze test (EPM)*** | | |
| Mice | Long-term | 3 |
|  | Short-term | 6 |
| Rats | Long-term | 1 |
|  | Short-term | 6 |
| ***Open field test (OF)*** | | |
| Mice | Long-term | 2 |
|  | Short-term | 5 |
| Rats | Long-term | 2 |
|  | Short-term | 6 |

Data were extracted from Supplementary Table 2

**Supplementary reference**

1. Altman DG, Machin D, Bryant TN, Gardner MJ. Statistics with confidence: Confidence intervals and statistical guidelines. *Journal of The Royal Statistical Society Series A-statistics in Society*. 1990;153:256-256.
